# Recruiting adult participants to physical activity intervention studies using sport: a systematic review

**DOI:** 10.1136/bmjsem-2017-000231

**Published:** 2017-07-11

**Authors:** Rachel Cooke, Andy Jones

**Affiliations:** Norwich Medical School, University of East Anglia, Norwich, UK

**Keywords:** Systematic review, Sport, intervention, recruitment, physical activity

## Abstract

**Objective:**

To undertake a systematic review of the effectiveness of recruitment mechanisms for engaging and retaining target participants in sports interventions to promote physical activity behaviour change in adults.

**Design:**

A narrative systematic review of published studies providing details of the effectiveness of recruitment techniques used in interventions aimed at increasing physical activity via sport in adults.

**Data sources:**

Searches were conducted using five electronic databases, clinical trial registers, grey literature and snowballing from reference lists. All papers published in the English language were considered. The search was completed in November 2015.

**Eligibility criteria:**

All articles providing information on the recruitment of adults into interventions involving sport and reporting physical activity or participation outcomes were included.

**Results:**

Twenty-three studies met the inclusion criteria. The quality of recruitment reporting across included studies was generally classified as poor, lacking detailed descriptions of recruitment processes and providing insufficient reporting of recruitment outcomes. There was a distinct recruitment bias for more affluent, white, middle-aged women. Active-only recruitment techniques appeared to achieve a participant sample with more representative demographic characteristics than passive approaches.

**Conclusions:**

Due to inadequate reporting and evaluation, the mechanisms for achieving effective recruitment and engagement in sport, particularly in hard-to-reach groups, are still unclear. Independent of recruitment mode, creating an intervention and context that reflect the interests and motivations of the target audience presents a promising area. There is an urgent need for more robust evaluation design and reporting of sports interventions.

**What are the new findings?**

- Evaluation and reporting of recruitment processes in sports interventions is scarce resulting in a lack of evidence for suitable engagement mechanisms, particularly in hard-to-reach groups.
- Active-only recruitment approaches achieved more representative samples for their target population than passive or combined techniques. It is of concern, however, that these approaches may be more vulnerable to limited participant engagement, thus requiring additional components such as motivational interviewing to encourage participation.

**How might it impact on clinical practice in the near future?**

For future sporting interventions, techniques involving active recruitment may be particularly appropriate to recruit hard-to-reach participants or to better achieve target participant demographic composition.

## Introduction

Physical inactivity is a global public health problem and the fourth leading cause of global mortality, resulting in over 5.3 million deaths a year worldwide.^1^ It costs £0.9 billion to the National Health Service in the UK alone.^2^ Sport presents a possible means of promoting physical activity (PA) and health. However, little is known about how best to engage inactive individuals in sport to increase PA.^3^

It is recognised that PA and sports participation are unequally distributed across society with gender, age, disability, education and socioeconomic status (SES) as determinants.^4^ Certain subgroups of the population are therefore more likely to be inactive and stand to gain significant health benefits from increasing their PA.^5^ These groups are frequently considered ‘hard to reach’, and as such, it is important to understand how they may be successfully engaged in health-promoting interventions.^3^ Nevertheless, historically there has been a significant recruitment bias in PA interventions that have predominantly recruited white, middle-class, middle-aged women, resulting in under-represented male, socioeconomically disadvantaged and minority ethnic populations.^6^ In light of this, sports interventions are becoming increasingly targeted in attempts to reduce health inequalities and engage underserved populations.^7^

In general, academic journals have prioritised the publication of intervention findings above the evaluation and reporting of recruitment processes and outcomes.^8 9^ This is limiting because, independent of intervention efficacy, the viability of a programme will be determined by its ability to recruit sufficient numbers of eligible participants.^10^ This limitation is further exacerbated by the fact that there has been a general lack of evaluation of sports programmes, thus limiting the evidence base on how to engage often hard-to-reach inactive populations in sport.^3 11^ We argue that a better understanding of recruitment procedures and their effectiveness is needed to inform those wishing to successfully replicate or adapt interventions, particularly when informing policy or practice.^12^ This review has therefore been undertaken to provide evidence for the role of planning, implementation and reporting of recruitment processes for sports interventions promoting positive PA behaviour change.

## Method

This review systematically identifies and evaluates the effectiveness of recruitment techniques used in interventions aimed at increasing PA using sport. Although the definition of recruitment varies between studies, we have defined ‘recruitment’ to be those who enrol on an intervention independent of whether they participate. We further define recruitment effectiveness as engaging sufficient numbers of target populations to (1) register for the intervention, (2) participate, (3) complete the intervention or any follow-up, and (4) achieve long-term positive PA behaviour change. Consequently, for the purpose of this review, retention is also considered a component of recruitment effectiveness. From a methodological perspective, the two primary challenges were the identification of reports on PA interventions using sport and the subsequent identification of the recruitment methods used. Hence, the adopted methodology was designed to particularly address these issues.

Inclusion criteria and analysis methodology were previously specified and documented in a protocol registered as CRD42015015815 (available at http://www.crd.york.ac.uk/PROSPERO/display_record.asp?ID=CRD42015015815).

### Data sources

To identify potential studies, searches were conducted using electronic databases, clinical trials registers, grey literature and snowballing from reference lists. This involved a systematic search of the following electronic databases: CINAHL, MEDLINE, EMBASE, PsycINFO and SPORTDiscus. Then the two primary organisations in England contributing to the delivery of sports programmes for PA and health (Sport England, the national body for sport in England, and UKActive, a not-for-profit health body for the PA sector in the UK) were contacted to identify additional grey literature. Titles and abstracts of identified material were checked against inclusion and exclusion criteria for suitability. Full articles were then acquired and assessed for inclusion. Snowballing was employed whereby, following inclusion, the reference lists of papers were searched and further articles considered for inclusion. The search was completed in November 2015.

The search terms used were developed using those of earlier systematic reviews on the evaluation of participation outcomes of sporting interventions^13^ and recruitment into walking interventions^10^ combined with an extensive list of sporting activities recognised by Sport England.^14^ Details of the search syntax used for electronic databases are provided in the online supplementary appendix. For clinical trials registers, ‘sport’ was the only search term within the title, using interventional studies in adults or seniors as limiters. The details of the full inclusion and exclusion criteria are provided in  online supplementary table 1. Pragmatic considerations meant the search was limited to papers published in English. All programmes recruiting adults into interventions involving sport and reporting PA or participation outcomes were included.

10.1136/bmjsem-2017-000231.supp1 Supplementary data

10.1136/bmjsem-2017-000231.supp4 Supplementary data

### Study selection

The first reviewer conducted a review of all identified studies to exclude duplicates and studies that clearly did not meet the inclusion criteria, for example, interventions looking at elite sport performance-related outcomes. To check the inclusion process, 15% of review articles were randomly selected at the abstract screening stage and screened by the second reviewer, and all papers were found to have been correctly excluded. Where duplicate studies presented, the journal article reporting the most recruitment data was analysed and other articles excluded unless multiple papers were found to report on distinctly different aspects of the intervention and still meet the inclusion criteria.

### Data extraction

A data extraction table was developed by both authors guided by the protocols of other reviews looking at recruitment into health interventions for guidance.^15–17^ The resultant table summarised the characteristics of the study, population, sport intervention, recruitment, retention and outcomes. Corresponding data were then extracted from all included papers by the first and second reviewers and were transcribed into the table using Microsoft Excel.

### Data synthesis

We anticipated considerable heterogeneity between identified interventions and their recruitment approaches and therefore planned to employ a narrative synthesis of results. Extracted information was also used to synthesise additional data relating to the study quality, efficiency and effectiveness following the methodology of a similar review.^10^

### Assessment of quality of recruitment reporting

Due to the specific focus of this review, we did not attempt to assess the general quality of studies but rather focused on the quality of reporting of recruitment. This was assessed using criteria previously developed by Foster *et al* 10 in their review of recruitment into walking intervention studies. Two reviewers independently assessed the quality of recruitment reporting in the studies regarding where the population was recruited, who conducted the recruitment, the time spent planning and preparing the recruitment, the time spent conducting the recruitment and the target population to be recruited. Each criterion was given a value of zero (absent or inadequately described) or one (explicitly described and present). Using the nomenclature of Foster *et al*,^10^ studies scoring three or below overall were considered ‘low quality’ while those that scored between four and five were considered ‘high quality’.

### Assessment of efficiency

We also adopted the methodology of Foster *et al* 10 to evaluate the efficiency of recruitment processes within included studies. This involved calculating the recruitment rates and efficiency ratios, where possible, for each included study. The following values were sought: the total number of potential participants who could be eligible for study (‘pool’), potential participants invited to participate in the study (‘invited’), potential participants who responded to the invitation (‘responded’) and participants who were assessed as eligible to participate and began the programme (‘started’). Where possible, ratios were calculated for each stage, for example, by dividing the number of participants who ‘started’ the study by the total ‘invited’ the proportion taking up the intervention. Furthermore, a weekly rate of recruitment was calculated for those studies also providing recruitment duration.

## Results

### Study characteristics

**Figure 1:**
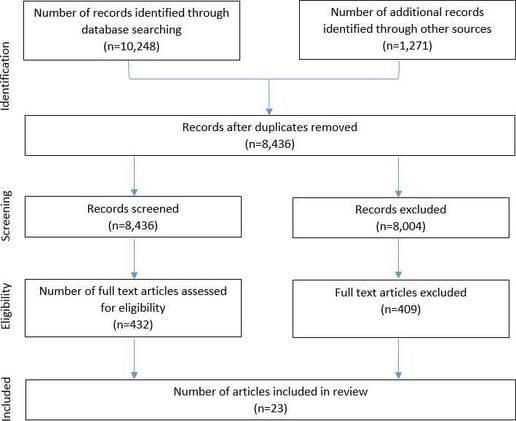
Review flow chart.

Twenty-three papers representing 22 interventions met our inclusion criteria. Figure 1 reports the flow of studies through the review process. Characteristics of included studies are presented in online supplementary table 2, ranked by quality score. Each included paper is referenced in the results and discussion sections in superscript using their reference citation. Full references for included papers are therefore listed in the bibliography. Studies were located in the UK (n=10),^18–27^ USA (n=5),^28–32^ Canada (n=3),^33–35^ Norway (n=2),^36 37^ Italy (n=1),^38^ Switzerland (n=1)^39^ and South America (n=1).^40^ Nearly all the studies were quantitative experimental studies in design, with 9 randomised controlled trials,^18 27–30 32 33 35 40^ 2 non-randomised controlled trials^25 37^ and 11 before-and-after studies.^19 21–24 26 31 34 36 38 39^ Of these, four were mixed methods and incorporated some qualitative design.^19 21 28 34^ We found only one qualitative study reporting on recruitment approaches.^20^

### Intervention characteristics

There were a wide variety of intervention designs and sports demonstrated across the included studies (see online supplementary table 3). Twelve interventions involved multiple physical activities, all or some of which involved participating in sport,^18–27 36 37^ while 11 offered an intervention using only one sporting activity.^28–35 38–40^ The main activities reportedly used, in isolation or combination, were dance (n=8),^23 25 29 32 34 37 38 40^ football (n=7),^18–21 23 26 37^ exercise classes (n=7),^19 24 25 27 31 37 38^ running/jogging (n=6),^18 23 24 33 38 39^ swimming (n=5)^23 24 36–38^ and yoga (n=2).^28 30^ A range of settings were used to deliver interventions, including professional football stadia and facilities,^18–21 26^ leisure or sports facilities^22 25 28 31 33 34^ and non-sporting community sites such as schools and churches.^27 29 36 38^ The average reported intervention duration was 23.7 weeks (SD ±27.7 weeks, range 8–104 weeks).

### Characteristics of the participants

Sample sizes (N started) of the studies ranged from 15 to 160 018 participants. Twenty studies reported participant ages^18–22 25 27–40^ with a mean age of 51.3 years (SD ±6.3 years) and range from 18 to 70 years (see online supplementary table 4). Six out of 22 studies that reported gender focused on recruiting female-only participants,^24 29 32 34 35 39^ and five studies recruited only men.^18 20 21 26 33^ From the remaining 12 studies that did not recruit sex-specific groups, 67% (SD ±16.2%) of participants were women. Thirteen studies reported ethnicity.^18–22 25 29–35^ Three studies reported targeting a single specific ethnic group: African-Americans,^29^ South Asians^34^ and ‘coloured’ (a term used by the authors) ethnicities.^32^ Of the remaining studies, 10 reported other ethnicity data; 80% of these participants were white Caucasian (SD ±16%, range 54%–100%).^18–22 25 30 31 33 35^ Sociodemographic data (SES or income groups, education) were not consistently reported. An area-based index of multiple deprivation was reported in two studies, one of which reported the highest proportion of participants in the two least deprived quintiles (5=25.1%, 4=22.2%)^18^ and the other in the two most deprived quintiles (1=25.7%, 2=20.29%).^27^ Average household income was below the poverty threshold in one study^26^ and indicative of a relatively high socioeconomic status in another.^32^ Seven studies reported employment status.^20 21 28 30 34 36 38^ In six of the seven studies, the majority of participants were employed.^20 21 28 30 34 36^

### Overview of recruitment reporting

Five studies were classified as ‘high’ quality^18 28 29 33 34^ and the remaining 18 classified as ‘low’ quality in relation to recruitment reporting (see online supplementary table 5). All studies reported the setting where the recruitment of participants took place, but eight did not report who conducted the recruitment.^25–27 32 36–38 40^ None of the included studies reported the time spent planning or preparing their recruitment, although six studies reported the time spent conducting recruitment.^18 25 28 29 33 34^ All studies detailed the target population for recruitment (see online supplementary table 8).

#### Recruitment data reported

Because none of the studies reported the time spent planning or preparing the recruitment, no studies covered all stages of the recruitment process. All of the studies reported a specific target group and some details of where recruitment was conducted, although these were often non-specific (see online supplementary tables 6 and 8). Most popular were community settings (n=14) such as community centres,^31 34^ sports clubs,^18–21 26^ places of worship^29^ and locally distributed advertising/media.^27 30^ Medical or care settings were also popular (n=6).^24 25 30 36 38 40^ Universities^22 35^ and workplaces were also used.^33 39^

Seventeen studies reported who conducted the study recruitment. Most frequently reported recruiters were research staff (n=5), which reflects the evaluative nature of much of the literature.^18 28 29 35 39^ Seven studies reported the time spent on implementing recruitment,^18 25 28 29 33 34 37^ which averaged as 52 weeks (SD ±71 weeks, range 4–156 weeks) (see online supplementary table 6).

#### Recruitment planning and implementation

The reporting of recruitment methods was inconsistent and varied across studies (see online supplementary tables 6 and 7). The exact number of recruitment methods used was generally not disclosed and difficult to infer from the recruitment description. Five studies relied on one method of recruitment only,^26 28 30 36 38^ while 14 studies used two or more approaches.^18 21 23–25 27–29 31–33 35 37 40^ Recruitment approaches were categorised as ‘passive’ or ‘active’. ‘Passive’ recruitment techniques prompt potential participants to identify themselves for the programme,^41^ whereas ‘active’ techniques require those involved in the programme to initiate contact with a potential participant (eg, health professional referrals).^42^ No relationship between the quality of recruitment reporting and the number of recruitment strategies used was observed. We did observe that while a number of studies used only passive techniques (n=6),^27 28 30 35 39 40^ most used a mixture of active and passive (n=13)^18–20 22–25 29 32–34 37^ and a small number used only active methods (n=3) (see online supplementary table 7).^31 36 38^

#### Recruitment rates and efficiencies

We were unable to extract all of the values for the ‘pool, invited, responded and started’ participation levels required to calculate efficiencies across the recruitment process as set out by the Foster *et al* 10 review (see online supplementary table 9). We were, however, able to calculate a weekly recruitment rate using the final number of participants divided by the time spent recruiting in weeks for six studies (mean 13 participants per week, SD ±14, range <1 to 37 participants per week). Only one study reported the volume of participant uptake grouped by recruitment method.^18^

### Physical activity outcomes

Physical activity outcomes were reported both directly, using validated measures of PA, and indirectly through the reporting of attendance or participation across the included studies (see online supplementary table 10). Of those studies reporting change in PA, 14 reported significant increases in PA between baseline and the end of the intervention.^18 20 21 25 28 29 32 33 35–40^ Only five of these, however, reported maintaining a significant increase in PA from baseline at post-intervention follow-up^32 36 38–40^ with four reverting to non-significant differences.^18 28 29 35^ Furthermore, one study reported a significant increase in PA at the post-intervention follow-up but not at the end of the intervention^30^ and another^27^ had no significant changes in PA to report at the end of the intervention. In the studies that did not quantify PA, records of attendance were used to represent PA participation during the intervention period.

### Recruitment target, exclusion and study retention

Sample size calculations were referenced in five studies,^18 27 29 36 37^ of which two indicated they were used to provide a target sample size for recruitment,^18 29^ which was successfully achieved by one^18^ and not the other.^29^ Fourteen studies indicated that there was a screening process to determine eligibility within the recruitment pathway.^18 23 26 28–32 34–36 38–40^ However, only five reported the proportion of recruits found to be ineligible at this stage (mean 18%, SD ±17.9%).^18 28 29 32 39^ The number of recruited participants who did not attend the intervention was reported in six studies (mean 19%, SD ±22.9%).^26 29 31 34 35 40^ The cost of recruitment per participant was calculated in one study at £20.32.^25^ It was not clear in the majority of studies whether incentives for recruitment (n=0) and retention (n=2)^18 31^ were offered. Attendance was reported in a variety of ways in 15 studies.^18 22 24–26 28 29 31–35 38–40^ The average reported attendance was 77% (n=9, SD ±12.4%, range 57%–100%).

Retention figures were reported in 16 studies.^18 19 21 23 25 26 29–31 33 35–40^ Retention reporting could either refer to participation in follow-up (study retention) or participation in PA or sport (PA retention) as part of the intervention or beyond. The average reported study retention rate at first follow-up was 82% (n=13, SD ±14.8, range 49–100),^18 19 21 26 29 30 33 35 36 38–40^ whereas the average reported PA retention rate was 28.5% (n=2, SD ±13.4, range 19–38).^23 31^ Number and reason for dropouts was reported in 11 studies.^18 25 26 29 30 33–36 39 40^ Most commonly cited reasons for dropout were illness or injury, work, unexpected commitments, lack of time, relocation or travel, and disliking the intervention.

### Additional comments reported regarding recruitment and retention

Comments relating to recruitment highlighted word of mouth^19 21 22 24 25^ and social media^22–24^ as valuable recruiters. Additionally, the role of recruitment partnerships^19 20 23 24^ as well as active,^24^ passive^25^ and multiple^23–25^ recruitment mechanisms were discussed. The design of promotional materials was highlighted in a number of studies.^22–24^ The intervention setting,^20 21^ appeal of the activity^18 19 21 22 25^ and opportunities for socialising^19 23^ were also important for recruitment of the target group. Several studies commented on the successes^20 21^ and challenges^24 27^ of reaching the target group.

Facilitators of retention discussed included social support,^19 21 25 26 34 38 39^ variety of activities,^19 21 22 24^ group cohesion,^34 39 40^ fun/enjoyment,^21 25 34^ coaching,^24 31^ routine,^19 21^ accessibility of delivery site,^21^ competition,^22^ timing of sessions,^24^ affordability,^24^ use of incentives,^24^ availability of progression opportunities,^24^ high programme satisfaction^34^ and higher baseline self-motivations towards PA.^39^ Barriers to retention included dropout or non-attendance in the early stages of the intervention,^18 25^ if individual activity intervention rather than group was used,^38^ the appeal of the activity^21^ and degree of competition.^21^

## Discussion

The effectiveness of any PA or sporting intervention is limited by the impact the intervention has on its participants and by the effectiveness of its recruitment of eligible participants to take part in the intervention.^10^ This systematic review showed that the evidence on how best to recruit is sparse due to the absence of generalisable findings and insufficient reporting of recruitment methodology and process outcomes in sports-based PA interventions. Furthermore, there is an absence of evidence linking specific recruitment methods to more successful long-term behaviour changes due to a lack of clarity surrounding recruitment channel outcomes. Lack of reported information meant we were also unable to asses the cost per person of recruitment for different recruitment strategies. This would be of particular interest as there is an inevitable cost–effect trade-off to be considered when designing recruitment mechanisms.

We know that reaching priority groups for PA studies, such as the inactive or unhealthy, socioeconomically disadvantaged, ethnic and other minority groups, is challenging.^9^ Our review demonstrates that current recruitment strategies engage predominantly white, middle-class, middle-aged women unless they are clearly designed to target specific demographic characteristics, such as gender or ethnicity. Furthermore, this recruitment bias for particular populations is supported by an earlier review of wider PA interventions.^6^ Our review also found that despite targeting some of these demographic characteristics successfully, interventions tended to achieve unrepresentative levels for the remaining untargeted characteristics such as socioeconomic status. For example, Vahabi and Damba^34^ recruited predominantly Indian women in a South Asian Bollywood dancing intervention. However, most of these women were in full-time employment and had a university degree or higher, suggesting they were of a relatively high socioeconomic status. These findings indicate that typical recruitment techniques adopted by sports PA interventions are not reaching those most in need. Furthermore, targeting specific isolated characteristics may not be sufficient to alleviate recruitment bias towards particular demographic characteristics, thus limiting the generalisability of findings for policy and practice.

The use of active-only recruitment techniques^31 36 38^ (whereby those involved in the study or programme make the first contact with a participant) appeared to achieve a participant sample with more representative demographic characteristics than passive approaches (ie, prompt potential participants to identify themselves) not targeting specific demographics. This finding is supported by Mutrie *et al*’s paper,^9^ which found that active techniques were less likely to encourage self-selection and introduce recruitment bias than passive techniques. For example, a recruitment and intervention partnership targeting low-income demographics via community health services (active)^31^ successfully represented at-risk populations (52% women, 54% white, average income less than 100% of the federal poverty level). However, this study had a 63% non-use attrition rate, thus demonstrating a low conversion to participation. This supports existing evidence that place-based strategies (ie, recruiting participants from a location where they already aggregate) result in more representative samples, but a lower overall participation rate.^9 43^ There is, however, evidence from the Mangeri *et al* paper^38^ that techniques such as motivational interviewing and offering group intervention activities may encourage higher levels of participation in actively recruited participants. These observations highlight the difficulties and tensions faced by sporting interventions for reaching those most at risk or need. Due to insufficient monitoring and evaluation of recruitment processes, we were unable to determine which specific methods were more effective at engaging particular populations.

### Setting

In addition to recruitment mode, the reach of a programme is determined by the appeal of characteristics of the intervention to target participants. Further, research processes such as having to complete questionnaires or wear an accelerometer may also influence uptake and impact recruitment to the programme in general. This review highlights the diversity and effectiveness of a subgroup of interventions delivered in professional football (soccer) clubs for engaging target populations in PA and health-promoting activities.^18–21 26^ The professional football context in which these programmes were delivered was reported as a powerful draw for participants.^19–21 44^ Furthermore, the range of populations successfully targeted and recruited by these interventions demonstrates the wide reach of professional sports settings. Using football club facilities, events, branding and media channels were all thought to contribute to the engagement of target groups, extending beyond the fan base of the host club.^45^ These programmes, therefore, highlight the importance of delivering interventions in contexts that are appealing to their target group and that use pre-existing interests and communities to achieve successful engagement.

### Reporting

The quality of recruitment reporting across included studies was generally classified as poor, with a distinct lack of information on how much time was spent planning and implementing recruitment. The majority of interventions reported using multiple, simultaneous recruitment methods, but only one reported the effectiveness of individual approaches.^18^ Instead, recruitment was largely generalised to a location or setting, thus making it unclear which method or combination of methods were most effective at recruiting particular target groups and achieving long-term behaviour change.

Higher level overviews of target populations were generally well reported. However, few studies described the pool of potential participants, the number of individuals invited to participate, how many responded and how many started the intervention, all of which were required to calculate recruitment efficiency in accordance with the Foster *et al* review.^10^ In fact, comparable levels of reporting were seen between studies included in our review and Foster *et al*’s,^10^ thus restricting both reviews’ ability to compute and compare efficiency ratios across included studies. Further to this, we also sought to investigate some process outcomes that are of interest when looking to translate research into practice: target sample size and the extent to which it was achieved, the eligibility and representativeness of recruited participants as well as their participation and retention in the intervention. Unfortunately, these were also poorly reported, meaning there is limited process-based evidence to inform recruitment designs that facilitate powered and representative sample sizes for rigorous evaluation.

The need for improved reporting highlighted in this review may in part be driven by a previous lack of agreement on a standard framework for recruitment reporting. In England, a standard evaluation framework (SEF) has recently been introduced to guide programmes to collect and evaluate information relating to PA interventions.^12^ The SEF advocates the monitoring and reporting of a range of recruitment variables as part of its essential criteria for evaluating PA interventions. These include method of recruitment, target population characteristics, measures of the flow of participants through the intervention and dates of crucial time points such as first point of contact and follow-ups. Following adequate execution and reporting of the essential recruitment information, such as those set out in the SEF, the future application of data extraction and scoring procedures attempted within this review would likely produce a greater insight into recruitment processes for sporting interventions aiming to promote PA. We are unaware of similar guidance in other international settings, although these may exist. The level of measurement and reporting observed in this review, however, suggests that standard evaluation of recruitment variables is not being used elsewhere. In the UK, the SEF is a relatively recent document having been published in 2012, and as such its impact may yet to be seen in the academic literature. This may also be the case for other similar guidance, yet if comparable guidance has been in place for longer, our findings raise the question of the extent of its adoption and application.

### Strengths and limitations of this review

The strength of this review is that it used protocols, previously shown to be successful in other reviews, to systematically identify and analyse 23 sport PA intervention papers using a comprehensive search strategy and extensive data extraction table. We also attempted to compute a set of metrics to describe recruitment efficiency that were informed by similar systematic reviews investigating sport or recruitment.

Regarding limitations, there are limited published or publicly available evaluations of sporting interventions published in English as well as a reluctance from editors to publish articles where recruitment is the prime focus.^8^ This restricted conclusions relating to a number of processes of interest for this review. In line with the objectives of this study, we assessed the quality of recruitment reporting as our risk of bias tool. An implication of this was that we did not assess the overall quality of the included study. The lack of rigorous recruitment monitoring and reporting meant that the quality of extracted information on recruitment was not enough to allow a meta-analysis to be taken. A further issue in this review was that a number of the programmes considered were designed as research studies and consequently include additional research processes otherwise absent in typical sports interventions. There is evidence that characteristics of the population recruited can be influenced by recruitment protocols, intervention and research design characteristics.^9^ Research protocols may therefore impact recruitment differently to unevaluated sports programmes or those with a lighter-touch evaluation. Consequently, the generalisability of our results was limited by the difficulties of separating the effect of the research and evaluation from overall programme recruitment. Furthermore, extracted values for participant outcomes were limited to only those who participated in the reported evaluation and may not be representative of the complete participant population.

## Conclusions

Overall, this review emphasises the need for robust evaluation design and reporting of sports intervention and recruitment processes to permit future evidence-based interventions. There is a growing evidence base for the benefits of sport for physical inactivity and health. However, due to inadequate reporting and evaluation, the mechanisms for achieving effective recruitment and engagement in sport, particularly in hard-to-reach groups, are still unclear. There is a notable tendency of sporting interventions to recruit white, more affluent, middle-aged women. Simply targeting isolated demographic characteristics, such as gender or ethnicity, appears insufficient to recruit a sample representative of the population for the remaining, untargeted characteristics. Combinations of active and passive methods were commonly used, yet active-only recruitment approaches achieved more representative samples for their target population. It is of concern, however, that active-only recruitment may be more vulnerable to limited participant engagement, thus requiring additional components such as motivational interviewing to encourage participation. Independent of recruitment mode, creating an intervention and context that reflect the interests and motivations of the target audience, such as local professional football facilities, presents a promising area.

10.1136/bmjsem-2017-000231.supp2

10.1136/bmjsem-2017-000231.supp3 Supplementary data
