# Supplementary material for: Recruiting adult participants to physical activity intervention studies using sport: a systematic review
Source: BMJ Open Sport Exerc Med. 2017 Jul 11;3(1):e000231. doi: 10.1136/bmjsem-2017-000231 (PMC5530105; doi:10.1136/bmjsem-2017-000231)
Supplement: Supplementary data [file bmjsem-2017-000231supp001.pdf]

Table 1. Inclusion and exclusion criteria

**Inclusion**	**Exclusion**
Adults (19 years and above).	Children and youths (up to and including 18 years).
The intervention includes a Sport England recognised sport as either a single sport or part of a range of sports and activities.	Interventions that do not involve a Sport England recognised sport.
Increasing physical activity is an aim and physical activity levels or participation are a measured and reported outcome of the intervention. This can include objectively or subjectively measured physical activity, attendance or participation.	Increasing physical activity is not an aim of the intervention, and therefore physical activity or participation is not a measured and reported outcome.
Where participants are recruited into an intervention that includes a sporting activity they were not already participating in.	Where participants are not recruited into an intervention that includes a sporting activity they are not already participating in. e.g. data collected on adults already taking part in sport, cohort studies.
Studies where the methods of recruitment are reported or are identifiable through correspondence with the authors.	Studies where details of the recruitment method are absent and unavailable on request from the authors.

Table 2. Characteristics of included studies

Study Number, Author and Pub. Year	Country	Study Type	Study Aim	Target Population	Recruitment Quality Score
Bryan, 2012[20]	USA	Pilot randomised control trial, Mixed methods	Assess the effects of 10 weeks of yoga classes held twice a week onphysical activity adherence in previously inactive adults.	Adult men and women, aged 18-65 years who were healthy but who categorised themselves as having been physically inactive for the previous 6 months.	4
Cunningham, 1987[21]	Canada	Randomised control trial	Describe the effects, of a year of regular physical activity, particularly running, on the cardiorespiratory fitness and health related factors in men at the time of retirement from regular employment.	Men, retiring from work.	4
Murrock, 2006[22]	USA	Cluster randomised waiting list control trial	Investigate the effectiveness of a culturally-specific dance intervention for increasing physical activity and functional capacity in sedentary African American women.	Sedentary African American women, ages 40 years and older.	4
Vahabi , 2015[23]	Canada	Before-and-after study, Mixed methods	Assess South Asian women's participation in Bollywood dance classes offered for a 6-week period, and examine the impact of Bollywood dance on physical, mental, and social health.	Inactive adult South Asian immigrant women.	4
Wyke, 2015[24]	UK	Waiting list randomised control trial	Evaluate the effectiveness of a weight loss, physical activity and healthy living programme for men aged 35–65 years with a BMI of at least 28 kg/m2, delivered in football clubs.	Men aged 35–65 years with a BMI of at least 28 kg/m2	4
Alexander, 2012[25]	USA	Longitudinal comparison between two randomised control trial's	To describe patterns of yoga practice and to examine differences in physical activity over time between individuals with or at risk for type 2 diabetes who completed an 8-week	Individuals with or at risk of type 2 diabetes.	3

Study Number, Author and Pub. Year	Country	Study Type	Study Aim	Target Population	Recruitment Quality Score
			yoga-based intervention, compared with controls.		
Boyd, 2006[26]	USA	Before-and-after study	Investigate the impact of a partnership with a community health centre to improve access to and participation in physical activity for low-income patients with type II diabetes.	Individuals over 19 with type 2 diabetes.	3
Jepson, 2015[27]	Norway	Before-and-after study	Investigate the impact of a physical activity intervention offering a range of activities on change in physical activity; physical, mental, and obesity-specific quality of life and life satisfaction in severely obese adults.	Severely obese adults.	3
Mangeri, 2014[28]	Italy	Two-arm before-and-after study	To test the effectiveness of a dance programme to improve fitness and adherence to physical activity in subjects with type 2 diabetes and obesity.	Adults with type II diabetes and/or obesity.	3
McDonoughn, 2010[29]	Canada	Waiting list randomised control trial	Evaluate the effect of a dragon boating intervention on psychosocial predictors of exercise and physical activity levels among overweight women.	Overweight women.	3
Parnell, 2015[30]	UK	Before-and-after study<br>Mixed methods	Examine the potential of professional football clubs as ‘community hubs’ for strongly engaging older adults in physical activity and health improvement activities.	Older adults aged 55+ years	3
Pfister, 2013[31]	Switzerland	Before-and-after study	Evaluate the effect and feasibility of a jogging training programme for increasing physical activity in inactive female hospital staff members.	Physically inactive female hospital staff members aged 45 and older.	3

Study Number, Author and Pub. Year	Country	Study Type	Study Aim	Target Population	Recruitment Quality Score
Pringle, 2013[32]	UK	Qualitative	Identify the key design characteristics in reaching and helping participants adopt health improvement interventions, including physical activity, delivered in/by the English premier league football clubs.	Men aged 18-35 years.	3
Pringle, 2014[33]	UK	Before-and-after study, Mixed methods	Using final impact data derived from the programme evaluation, this study set out to assess the effect of the overall programme on health outcomes, including physical activity, along with men's experiences from participating in interventions.	Men aged 18-35 years.	3
Sport England, 2014b[34]	UK	Before-and-after study, service evaluation	Evaluate the impact of the programme on University student participation in sport activities.	University students.	3
Sport England, 2014c[35]	UK	Before-and-after study, service evaluation	Investigate the preliminary impact of a range of interventions targeting inactive adults to increase physical activity through participation in sport.	Inactive individuals, 14 years and above. Individual programmes may also target other factors such as medical conditions, demographic or health risk groups.	3
Vivian, 2014[36]	UK	Before-and-after study, service evaluation	Gain an understanding of the efficacy and value of the programme for encouraging participation in sport among women.	Women, particularly those living in deprived areas or caring for children.	3
Withall, 2012[37]	UK	Non-randomised control trial	Examine the effect of a social marketing campaign on the monthly recruitment, attendance and retention levels at a community-based physical activity programme in a low income area.	Individuals with an awareness of the benefits of exercise and positive attitudes towards it but with anxiety-related barriers to joining exercise sessions.	3
Brady, 2010[38]	UK	Before-and-after study	Evaluate the sustained impact of a health project for middle-aged football supporters on diet and exercise.	Men aged 40-60 with a high BMI.	2

Study Number, Author and Pub. Year	Country	Study Type	Study Aim	Target Population	Recruitment Quality Score
Foster, 2013[39]	South America	Randomised control trial	Evaluate the effectiveness of a 12-month community-based tango dance programme on activity participation among individuals with Parkinson disease.	Adults with Parkinson disease.	2
Lee, 2011[40]	USA	Cross-over randomised control trial	Determine whether a Latin dance intervention was sufficient to promote increase in weekly physical activity in women of colour.	Women of colour.	2
Lupton, 2003[41]	Norway	Cluster non-randomised controlled trial	Evaluate the long-term lifestyle and risk factor changes occurring after a three-year community-based intervention, with a physical activity component offering a range of sports, in the municipality of Båtsfjord, Finnmark, North Norway, compared with three similar control communities.	Population of Båtsford.	2
Solomon, 2014[42]	UK	Stepped wedge cluster randomised control trial	Evaluate the effectiveness of a community-level physical activity intervention.	Residents of selected rural villages/regions of Devon, south-west England.	2

Table 3. Characteristics of study intervention

Study Number, Author and Pub. Year	Description of sport intervention	Intervention delivery site	Type of sport	Intervention duration
Bryan, 2012[20]	Hour-long hatha yoga classes that met twice a week for 10 weeks.	Yoga studio in central New Jersey.	Hatha Yoga	10 weeks
Cunningham, 1987[21]	**Intervention:** three group sessions per week, consisting of a 10- to 15-min warm-up, approximately 30 min of walking/jogging at an individualised pace, set to elicit the personal target heart rate range and a 10-min warm-down for one year after retirement.<br><br>**Control:** no intervention.	Exercise sessions were held outdoors on a 400m track during moderate and warm temperatures and on an indoor 200m track during poor weather conditions.	Running/ jogging	1 year
Murrock, 2006[22]	**Intervention:** Culturally-specific dance intervention led by an experienced African American female dance instructor, twice a week for 8 weeks, for a total of 16 sessions. Each dance session lasted for 45 minutes and included a 5-minute warm-up period; 30-minute dance segment, consisting of simple culturally-specific dance steps; and 10-minute cool-down period.<br><br>**Control:** waiting list site	Church	Dance	8 weeks
Vahabi , 2015[23]	6-week, twice weekly, Bollywood Dance exercise programme led by a female South Asian instructor. Each dance session lasted for 1 hour and included a 5-minute warm-up period, 45-minute dance segment, and a 10-minute cool-down period.	Sports centre or instructors dance studio	Bollywood dancing	6 weeks
Wyke, 2015[24]	**Intervention:**<br>12 weeks of once weekly 90 min session of health and diet advice combined with physical activity. Two physical activity components: 1) an incremental pedometer-based walking programme and 2) pitch-side physical activity sessions led by club community coaching staff to increase fitness through structured activities. These included aerobic (e.g. walking, stair climbing, jogging, football), muscle strengthening (e.g. weight/circuit	Football club home stadium	Multiple activities including sporting activities such as football and jogging	12 weeks

Study Number, Author and Pub. Year	Description of sport intervention	Intervention delivery site	Type of sport	Intervention duration
	training) and flexibility (e.g. warm-up/cool-down activities) exercises.<br><br>**Control:** 12 month waiting list			
Alexander, 2012[25]	**Intervention:**<br>Two 90 minute group sessions of supervised Iyengar yoga instruction per week, in addition to expected home practice, over an 8-week period. Iyengar yoga is a gentle activity, focusing on body alignment, postures and breathing exercises and includes the use of props such as blocks, belts, blankets, and chairs, all of which offer additional support for yoga positions to prevent strain or overstretching.<br><br>**Control:**<br>WHYS study: twice weekly, group educational meetings and discussion around diabetes.<br>DAYS study: usual care.	Not specified	Iyengar yoga	8 weeks
Boyd, 2006[26]	Patients received an orientation including a tour of the facility, an introduction to exercise equipment, individual goal setting, and a training class to assess the patients' current exercise tolerance level. All patients received free exercise classes, land or water aerobics, twice per week lasting up to 12 months. Patients also received a free 3-month YMCA membership and were eligible for a reduced membership rate thereafter.	Community sports centre	Exercise class - aerobics	1 year
Jepson, 2015[27]	Patients spent a total of 15 weeks at the rehabilitation centre divided into four stays over two years. The group-based cognitive behavioural therapy, consisting of eleven sessions over two years, targeted quality of life and self-management of physical activity and eating. Scheduled physical activity in the residential periods consisted of brisk walking, swimming, strength training,	Rehabilitation centre	Multiple activities including sporting activities such as; swimming, ball games and aerobics.	2 years

Study Number, Author and Pub. Year	Description of sport intervention	Intervention delivery site	Type of sport	Intervention duration
	ball games, and aerobics and amounted 9-11 hours weekly divided into bouts of 20–60 minutes. Each patient developed a plan for physical activity, modified to his or her preferences, limitations, and home situation.			
Mangeri, 2014[28]	**Dance programme**: Two-hour dancing session twice a week, led by instructors in a private hall of a disco. Both solo, partner and group dances were performed during each session: an initial one-hour activity, chaired by two instructors who taught new steps and choreographies to patients (both individually and in group), was followed by one hour dancing in pairs (Latin and standard ballroom music).<br>**Self-selected activity:** consisted of a wide variety of activities. The preferred activities were walking (36 cases), cycling (4 cases), swimming (6 cases), gym sessions (5 cases - with the support of sports associations), or home exercising (exercise bike, 6 cases), but also included bouts of mountain walking, golf, weight lifting, jogging, dancing. Such activities were recorded in a diary, also reporting time, duration and average speed, without external confirmation.	Private hall of a disco	Dance or self-selected activities such as walking, cycling, swimming, gym etc.	6 months
McDonoughn, 2010[29]	70 minute sessions of dragon boating, twice weekly for 12 weeks, involving skill instruction and intermittent aerobic and anaerobic exercise based on recommendations for novice dragon boat paddlers.	Not specified	Dragon boating	12 weeks
Parnell, 2015[30]	Club-based interventions varied between delivering clubs. Generally, activities consisted of 2 hour-long weekly classes and groups involving physical and social activities delivered free to participants. A broad menu of PA opportunities were offered, including exercise to music, indoor bowls, cricket, new age curling, walking football, alongside traditional board games, bingo, table tennis, zumba and skittles.	Football club facilities and community venues.	Football was one of the activities available in 14 of the 20 delivering clubs.	Not specified

Study Number, Author and Pub. Year	Description of sport intervention	Intervention delivery site	Type of sport	Intervention duration
Pfister, 2013[31]	3-month step-up jogging training administered by physical therapists twice a week. Progressively increased running intervals each week. Each training session included a practical and a theoretical part.	Not specified	Running/Jogging	12 weeks
Pringle, 2013[32]	Interventions in the programme were formed of either match-day events (n=12) and/or weekly classes and groups (n=16). A programme of regular weekly classes and groups for men. Activities were designed to improve the health of male recruits through interventions delivered in football club facilities and community venues. Often this involved playing football, but this was not universal and included other forms of PA combined with health promotion interventions.	Football club facilities and community venues.	Multi-sports. Football and fitness.	Not specified/Varied
Pringle, 2014[33]	Interventions in the programme were formed of either match-day events and/or weekly classes and groups. A programme of regular weekly classes and groups for men. Activities were designed to improve the health of male recruits through interventions delivered in football club facilities and community venues. Often this involved playing football, but this was not universal and included other forms of PA combined with health promotion interventions.	Football club facilities and community venues.	Football, gym-based activities, keep fit, circuits, badminton and walking, among other activities.	Not specified/Varied
Sport England, 2014b[34]	20 - 30 different sports.	Multiple. University setting	A range of Sport England recognised sports	Not specified/Varied
Sport England, 2014c[35]	The projects provide access to a range of informal and formal sporting opportunities that have been developed to support inactive people into sport. Sports delivered to date include swimming, running, cycling, boxing, netball, archery, table tennis, athletics, gymnastics, rugby, football, climbing, fitness dance, yoga etc. Ten of the projects have an element of motivational interviewing/counselling within them completed by health	Multiple	A range of Sport England recognised sports including; swimming, running, cycling, boxing, netball,	Not specified/Varied

Study Number, Author and Pub. Year	Description of sport intervention	Intervention delivery site	Type of sport	Intervention duration
	professionals, physical activity professionals or trained community members.		archery, table tennis, athletics, gymnastics, rugby, football, climbing, fitness dance, yoga etc	
Vivian, 2014[36]	A range of women-only sporting and physical activities e.g. netball, cycling, fitness classes, zumba. Projects offered both activities that could be done individually as well as team sports in order to appeal to a broad range of women.	Multiple	Sports varied between programmes. Particularly successful activities included badminton, running and swimming.	Not specified/Varied
Withall, 2012[37]	**Intervention:**<br>Three kinds of dance session (Line Dancing, Zumba and Salsa), an instructor-led gym session, and Body Tone, a balance and stretching session. These five sessions each took place once per week at 9.15 am, so a different session ran every weekday morning.<br><br>**Control:**<br>Control communities received no additional classes to what was already locally available.	Local leisure centres	Dance and exercise classes	6 months
Brady, 2010[38]	Weekly 2 hour sessions for 10 weeks, at local football stadium consisting of one hour of health discussion and an hour split into 20 min pitch side exercise; 20 min cardiovascular workout using treadmills and other aerobic equipment, and 20 min dietary advice and discussion.	Local football stadia	Multi-sports. Football and fitness.	10 weeks

Study Number, Author and Pub. Year	Description of sport intervention	Intervention delivery site	Type of sport	Intervention duration
Foster, 2013[39]	**Intervention:**<br>One-hour dance classes two times per week for 12 months. Dance classes were taught by an experienced dance instructor who was trained and supervised by the principal investigator to ensure appropriateness and safety for individuals with Parkinsons disease. The classes consisted of progressive Argentine tango lessons in which participants learned a new step in each class.<br><br>**Control:**<br>Usual care	Not specified	Argentine Tango	12 months
Lee, 2011[40]	**Intervention:**<br>Biweekly Latin dance group consisting of eight one hour Latin dance lessons, where they learned four basic Latin dance steps (salsa, merengue, bachata and cha cha) taught by a professional dance instructor who instructed the group together to music. Each session consisted of a review of the basic steps, variations on the steps, and a dance incorporating the steps. 5-10 minutes cool down, consisting of slow dance steps, stretching and guided breathing.<br><br>**Control:**<br>Internet-based dietary education intervention. Received 4 weeks of delivery and then crossed over to dance group and vice versa.	Not specified	Salsa dancing	8 weeks
Lupton, 2003[41]	**Intervention:**<br>A range of sport and physical initiatives introduced in the area as part of a bid to improve health. This included workplace volleyball and football tournaments. Regular organised dances. Condition specific swimming groups. Aerobics classes for ladies, physical training for individuals with heart disease, badminton groups for grown-ups, development of cycle trails, and ski tracks.	Multi-site	Mixed sports including aerobics classes, badminton, cycling and skiing	Not specified/Varied

Study Number, Author and Pub. Year	Description of sport intervention	Intervention delivery site	Type of sport	Intervention duration
	**Control:**<br>No intervention			
Solomon, 2014[42]	**Intervention:**<br>Intervention villages provided with 12 weeks of physical activity opportunities for all age groups, including at least three different types of activities per village. Each village received an individually tailored intervention, incorporating a local needs-led approach. Support was provided for a further 12 months following the intervention. Typically, activities were offered on a weekly basis over the twelve-week period. The activity sessions were subsidised using intervention funds. Delivery partners coordinated the intervention by finding suitable activity venues, purchasing necessary equipment, and hiring local experts to deliver the activities.<br><br>**Control:**<br>No intervention	Primary and secondary schools, sports clubs, and other partnerships	Each village received at least three different types of activities (e.g., basketball for primary school children, multi-sports sessions for adolescents, and fitness classes for adults).	12 weeks

Table 4. Characteristics of participants

Study Number, Author and Pub. Year	Mean age, SD or Range	Gender (% Female)	Ethnicity	SES/Income	Education
Bryan, 2012[20]	51 (range 34-65)	81.50%	Not specified	Employed 85.2%	Not specified
Cunningham, 1987[21]	62.7 (range 54-68)	0%	Caucasian 100%	Mix of blue and white collar job classification	Not specified
Murrock, 2006[22]	Intervention participants 58.05 (±10.18)<br>Control participants 59.43 (±11.9)	100%	African American 100%	Not specified	Not specified
Vahabi , 2015[23]	42 (range 22-58)	100%	Indian 78%	Majority of participants were in full-time employment	85% had a university degree or higher
Wyke, 2015[24]	47.1 (±8.0)	0%	White 98%	Scottish index of multiple deprivation;<br>1 - 17.5% (most deprived)<br>2 - 17.5%<br>3 - 16.4%<br>4 - 22.2%<br>5 - 25.1% (least deprived)<br>Missing 1.3%	No qualifications 9.5%; standard grades or highers 32.3%; vocational or HNC/HND 32.1%; university education 20.9%; Other 5.2%
Alexander, 2012[25]	59.5	88%	Caucasian 79%	68% currently working	68% 4 years of college or more
Boyd, 2006[26]	89% 40+ years	52%	Caucasian 54%	Average income that was less than 100% of the federal poverty level	Not specified
Jepson, 2015[27]	43.6 (±9.4)	75.50%	Not specified	Married/cohabiting 63%; Have children 55%; Employed 84%	College / University education 50%
Mangeri, 2014[28]	59 (±9; range, 41-70)	48%	Not specified	**Dance group:**<br>40% employed<br>**Self-selected activity group:**<br>54% employed	**Dance group:** Primary (62%), Secondary (error), Vocational (57%), Degree (12%). **Self-selected activity group:** Primary (29%),

Study Number, Author and Pub. Year	Mean age, SD or Range	Gender (% Female)	Ethnicity	SES/Income	Education
					Secondary (81%), Vocational (41%), Degree (21%).
McDonoughn, 2010[29]	42.44 (±11.82)	100%	Caucasian 83%	Not specified	Highly educated, 75% having at least a university undergraduate degree
Parnell, 2015[30]	Overall, 5.3% of participants were 50–54 years old, 59.9% were 55–74 and 34.8% were 75+.	60%	White British 95%	Not specified	Not specified
Pfister, 2013[31]	53.15 (±4.24)	100%	Not specified	Not specified	85% of participants have university level education
Pringle, 2013[32]	57% 18-34 years	0%	White British 70%	61% employed	Not specified
Pringle, 2014[33]	57% 18-34 years	0%	White British 70%	61% employed	Not specified
Sport England, 2014b[34]	16-19 (39%); 20+ (61%)	43%	White 63%; BME 37%		University students
Sport England, 2014c[35]	Not specified	Not specified	Not specified	Not specified	Not specified
Vivian, 2014[36]	Not specified	100%	Not specified	Not specified	Not specified
Withall, 2012[37]	60% 18-44 years	95%	Caucasian 76%	Not specified	Not specified
Brady, 2010[38]	Not specified	0%	Not specified	Not specified	Not specified
Foster, 2013[39]	69	58%	Not specified	Not specified	Not specified
Lee, 2011[40]	41.0 (±5.3)	100%	Coloured 100%	Mean household income $90,000 for a family of 4 suggesting a relatively high SES	74% college graduates

Study Number, Author and Pub. Year	Mean age, SD or Range	Gender (% Female)	Ethnicity	SES/Income	Education
Lupton, 2003[41]	**Intervention:**<br>Males 47.7<br>Females 47.5 **Controls:**<br>Males 48.6<br>Females 47.9	**Intervention:**<br>65%<br>**Controls:**75%	Not specified	Not specified	Not specified
Solomon, 2014[42]	58.7 (±15.3)	61%	Not specified	Indices of multiple deprivation:<br>1 – 25.7% (most deprived)<br>2 – 20.9%<br>3 – 19.8%<br>4 – 17.8%<br>5 – 15.8% (least deprived)	**Intervention group education age:**<br>≤18 62.3%<br>≥19 37.7%

Table 5. Assessment of quality of reporting recruitment

Study Number, Author and Pub. Year	Did the study report where the population was recruited?	Did the study report who conducted the recruitment?	Did the study report the time spent planning/ preparing the recruitment?	Did the study report the time spent conducting the recruitment?	Did the study target a specific population?
Bryan, 2012[20]	Yes	Yes	No	Yes	Yes
Cunningham, 1987[21]	Yes	Yes	No	Yes	Yes
Murrock, 2006[22]	Yes	Yes	No	Yes	Yes
Vahabi , 2015[23]	Yes	Yes	No	Yes	Yes
Wyke, 2015[24]	Yes	Yes	No	Yes	Yes
Alexander, 2012[25]	Yes	No	No	No	Yes
Boyd, 2006[26]	Yes	Yes	No	No	Yes
Jepson, 2015[27]	Yes	Yes	No	No	Yes
Mangeri, 2014[28]	Yes	No	No	No	Yes
McDonoughn, 2010[29]	Yes	Yes	No	No	Yes
Parnell, 2015[30]	Yes	Yes	No	No	Yes
Pfister, 2013[31]	Yes	Yes	No	No	Yes
Pringle, 2013[32]	Yes	Yes	No	No	Yes
Pringle, 2014[33]	Yes	Yes	No	No	Yes
Sport England, 2014b[34]	Yes	Yes	No	No	Yes
Sport England, 2014c[35]	Yes	Yes	No	No	Yes
Vivian, 2014[36]	Yes	Yes	No	No	Yes
Withall, 2012[37]	Yes	No	No	Yes	Yes
Brady, 2010[38]	Yes	No	No	No	Yes
Foster, 2013[39]	Yes	No	No	No	Yes
Lee, 2011[40]	Yes	No	No	No	Yes
Lupton, 2003[41]	Yes	No	No	No	Yes
Solomon, 2014[42]	Yes	No	No	No	Yes

Table 6. Recruitment planning and implementation (Quality metric categories)

**Study Number, Author and Pub. Year**	**Where did the recruitment take place?**	**Who did the recruitment?**	**Time spent planning/ preparing recruitment**	**Time spent executing recruitment (Weeks)**	**Population targeted (Yes/No)?**
Bryan, 2012[20]	In the vicinity of the participating yoga studio	Research team	Not specified	4 weeks	Yes
Cunningham, 1987[21]	Recruited through their company's personnel office, newspaper advertisements, and by word of mouth.	Company personnel office in part.	Not specified	156 weeks	Yes
Murrock, 2006[22]	During/after Sunday church service and advertised at the church.	The church. A respected woman from each church congregation, chosen by her minister. Research team members also attended the church services.	Not specified	4 weeks	Yes
Vahabi , 2015[23]	Community health centre	Community health centre personnel and eligible participants.	Not specified	8 weeks	Yes
Wyke, 2015[24]	In football clubs and club related settings	Research team	Not specified	10 weeks	Yes
Alexander, 2012[25]	Advertising in community newspapers, university buildings, community venues, and medical offices.	Not specified	Not specified	Not specified	Yes
Boyd, 2006[26]	Community health centre	Physician, physician assistant, advanced nurse practitioner	Not specified	Not specified	Yes
Jepson, 2015[27]	GP surgeries	GP referral	Not specified	Not specified	Yes

Study Number, Author and Pub. Year	Where did the recruitment take place?	Who did the recruitment?	Time spent planning/ preparing recruitment	Time spent executing recruitment (Weeks)	Population targeted (Yes/No)?
Mangeri, 2014[28]	From two diabetes/metabolic units	Not specified	Not specified	Not specified	Yes
McDonoughn, 2010[29]	University campus and in the local community	Research team	Not specified	Not specified	Yes
Parnell, 2015[30]	Posters, flyers, existing contacts and club media channels, such as match day programmes, the club website and magazines Advertisements in local press. Outreach work where older adults congregated (including local health centres) and via community leaders. Word of mouth was considered central to recruitment.	Varied across programme locations. Generally those who engaged captive older adult audiences.	Not specified	Not specified	Yes
Pfister, 2013[31]	Participants were recruited among female staff members of the University Hospital in Zurich, Switzerland	Research team	Not specified	Not specified	Yes
Pringle, 2013[32]	Match days, local groups, public services, local champions, informal networks	Health trainers, members of the delivery team, enthusiastic participants as champions	Not specified	Not specified	Yes
Pringle, 2014[33]	Varied across programme locations. Included a variety	Varied across programme locations.	Not specified	Not specified	Yes

Study Number, Author and Pub. Year	Where did the recruitment take place?	Who did the recruitment?	Time spent planning/ preparing recruitment	Time spent executing recruitment (Weeks)	Population targeted (Yes/No)?
	of locations in the local community across the different delivery sites. E.g. promotional events at clubs, local job centres. raising exercises delivered on match days to the supporters,	Generally partners who engaged captive male audiences			
Sport England, 2014b[34]	Programme coaches, volunteers and ambassadors at the University	Coaches, volunteers and ambassadors	Not specified	Not specified	Yes
Sport England, 2014c[35]	Varied across programme locations:<br>- 8 projects utilise self-referral<br>- 10 utilise referrals/ signposting from health professionals<br>- 4 utilise referrals/ signposting from other sources such as slimming clubs, community organisations etc.	Varied across programme locations: Health professionals, partners who engaged captive target audiences etc.	Not specified	Not specified	Yes
Vivian, 2014[36]	A range of locations, GP practices, partner organisations	A range of people. Ambassadors, GP's, partner organisations	Not specified	Not specified	Yes
Withall, 2012[37]	In the community via word of mouth, leaflets, local press, taster sessions,	Not specified	Not specified	25 weeks	Yes

Study Number, Author and Pub. Year	Where did the recruitment take place?	Who did the recruitment?	Time spent planning/ preparing recruitment	Time spent executing recruitment (Weeks)	Population targeted (Yes/No)?
	community groups, schools and GP's.				
Brady, 2010[38]	From Rangers and Celtic season ticket holders' database	Not specified	Not specified	Not specified	Yes
Foster, 2013[39]	Clinical research database	Not specified	Not specified	Not specified	Yes
Lee, 2011[40]	Brochures, word of mouth and local electronic newsletters	Not specified	Not specified	Not specified	Yes
Lupton, 2003[41]	Local community	Not specified	Not specified	156 weeks	Yes
Solomon, 2014[42]	Local press (newspapers, newsletters, and radio), and with posters in local sports centres and village halls.	Not specified	Not specified	Not specified	Yes

Table 7. Recruitment planning and implementation (Quality metric categories)

Study Number, Author and Pub. Year	No. of methods used	Procedures including who conducted the recruitment, where it took place and what was done	Active, passive or a mixture of approaches
Bryan, 2012[20]	1	Flyers written in English that the research team had placed in the vicinity of the participating yoga studio for a period of 1 month, from mid-October through mid-November.	Passive
Cunningham, 1987[21]	3	The men were admitted to the study over a 3-year period and were recruited through their company's personnel office, newspaper advertisements, and by word of mouth.	Active/Passive
Murrock, 2006[22]	3 - verbal announcement, information display table manned by research team and bulletin board announcement	Respected woman from each congregation, chosen by her minister to serve as a liaison, delivered a public announcement during Sunday church service that provided a verbal description of the study and its benefits and risks. In addition, the research study was advertised weekly in the church bulletin	Active/Passive
Vahabi , 2015[23]	2 - health centre recruitment and word of mouth	A convenience sample of 30 adult South Asian women was recruited either from selected community health centres that served the South Asian community, or through snow-ball technique. The study was advertised through posters and flyers at selected community health centres. Potential participants, who were either referred by the community health centre personnel or self-referred, contacted the research assistant. After assessing eligibility and explaining the study and process, the RA obtained signed consent from the women and set a convenient time and place for the first face-to-face interview. Participants who agreed to take part in the study were also asked to identify additional eligible women who may be interested to take part in our study.	Active/Passive
Wyke, 2015[24]	Multiple	Participants were recruited through club-based activities, media coverage, workplace advertising and word of mouth. **Club-based activities;** Posters/flyers with endorsement from club personalities; end of season home match advertising; active involvement of local supporters' organisations. Online publicity including Scottish Premier League website, club websites, fan websites, some club e-newsletters. **Publicity in media;** local and national newspapers; TV and radio coverage; workplace advertising; local councils and	Active/Passive

Study Number, Author and Pub. Year	No. of methods used	Procedures including who conducted the recruitment, where it took place and what was done	Active, passive or a mixture of approaches
		other employers; Other; Football Fans in Training (FFIT) Facebook page, FFIT website (www.spl-ffit.co.uk), FFIT participant online diary February to June.	
Alexander, 2012[25]	1	Participants for the DAYS and WHYS were recruited through advertisements posted in community newspapers, University buildings, community venues, and medical offices. These advertisements sought individuals who were interested in participating in a free yoga-based study.	Passive
Boyd, 2006[26]	Multiple	All patients were recruited and referred by providers (physician, physician assistant, advance nurse practitioner) from the Siouxland Community Health Centre (SCHC). During their SCHC provider visit, patients were given an exercise invitation similar to an exercise prescription with the address, directions, times, dates, and a brief description of the class.	Active
Jepson, 2015[27]	1	Referral of patients was done by general practitioners in accordance with the right to admission to the Norwegian specialist health services (i.e., BMI ≥ 40 kg/m2 or ≥ 35 kg/m2 with comorbidities).	Active
Mangeri, 2014[28]	1	During a recruitment session carried out according to the principles of motivational interviewing, the patients were asked to select two different physical activity options: a) a standard ballroom and Latin dance programme (DP) organized twice a week by diabetes associations, or b) a self-selected programme (SSAP), with the option to receive support from sport associations, for training sessions to be carried out at least twice weekly.	Active
McDonoughn, 2010[29]	1 - Posters	Participants were recruited through a poster campaign around the University campus and surrounding community. Posters were placed in a variety of visible, high-traffic locations including bus stops, grocery stores, food courts, community centres, libraries, and fitness facilities. Women who were eligible to participate were encouraged to call or email the researchers if they were interested in the project.	Passive
Parnell, 2015[30]	Multiple	Posters, flyers, existing contacts and club media channels, such as match day programmes, the club website and magazines were used to recruit. Advertisements were also placed in the local press and free newspapers. In some clubs, outreach work was undertaken where older adults congregated	Active/Passive

Study Number, Author and Pub. Year	No. of methods used	Procedures including who conducted the recruitment, where it took place and what was done	Active, passive or a mixture of approaches
		(including local health centres) and where community leaders were willing to help raise awareness of the programme. 'Word of mouth', whereby participants would tell their friends, was considered central to recruitment.	
Pfister, 2013[31]	1 - letters	Two-stage recruitment procedure. 4090 female employees worked at the University Hospital Zurich, 1249 of them were older than 45 years. 1) Contacted all 1249 female employees aged 45 and older with a letter asking about their level of physical activity, their motivation to become more active and willingness to participate in a physical exercise programme (assessed with four screening questions.) 2) Those who were classified as inactive were then invited to participate in the intervention. Those who agreed to participate were screened according to the inclusion criteria for the study to ensure suitability/eligibility (no significant musculoskeletal, cardiac or respiratory disorders).	Passive
Pringle, 2013[32]		Interventions delivered on match days were aimed at raising awareness of the programme. Health trainers (HTs) utilised local groups and public services offering potentially captive male audiences. HTs recruited enthusiastic participants to promote PLH to friends and reassure suspicious potential new recruits. Informal networks, such as word of mouth.	Active/Passive
Pringle, 2014[33]	3+	Recruitment followed (I) match day activities (II) outreach work with groups of local men such as workplaces, pubs, working men's clubs, mosques (III) club media e.g. website, club ambassadors (IV) local press (V) word of mouth/friends. Some groups started new recruits every 12 weeks supported by follow-on programmes. Recognising that new participants needed a group to join, some clubs operated rolling recruitment, meaning that old and new recruits were in the same sessions.	Active/Passive
Sport England, 2014b[34]	Multiple	Word of mouth communication, peer encouragement, social media, emails and printed posters were used across projects. Promotional and viral videos were also mentioned, as were pop-up stalls and taster opportunities in high footfall locations.	Active/Passive

Study Number, Author and Pub. Year	No. of methods used	Procedures including who conducted the recruitment, where it took place and what was done	Active, passive or a mixture of approaches
Sport England, 2014c[35]	Multiple	The projects utilise a range of recruitment methods to develop the evidence base with regards how to effectively engage and support inactive people into sport:<br>- eight projects utilise self-referral within their recruitment<br>- ten utilise referrals or signposting from health professionals (Primary Care and Public Health);<br>- four utilise referrals or signposting from other sources such as slimming clubs, community organisations etc.	Active/Passive
Vivian, 2014[36]	Multiple	Utilising ‘ambassadors’ / ‘champions’ where possible – these were women of the target type (from a deprived area or caring for children under the age of 16) who regularly took part in sessions or who had become further involved with their local Active Women project, whose role was to speak to women throughout the community face-to-face and encourage them to attend. Promotion of the active women projects specifically through non-sporting channels, for example through leaflets distributed at schools, as well as in shopping centres, GP surgeries, libraries and religious gatherings. Where resources allowed, projects put on demonstrations of certain activities, for example Zumba, netball, badminton, in public places such as shopping centres and school playgrounds, to emphasise the project’s focus on friendliness and fun, and to show the sessions as non-threatening and with a lack of emphasis on ‘sport’ and fitness. Programme, projects also used social media such as Facebook and Twitter, as well as making use of partnership working with other organisations to increase numbers. Examples included links with children’s centres who promoted the sessions amongst parents Projects also tried to ensure they provided easy-to-access practical information on sessions, such as eye-catching posters, leaflets and online. Much of the promotional material was designed to appeal to women who were likely to be relatively inactive and potentially uncomfortable in a sporting environment.	Active/Passive
Withall, 2012[37]	Multiple	A 6-month, social marketing campaign costing £7,395, delivered between late September 2010 and March 2011. The key promotional elements included 17	Active/Passive

Study Number, Author and Pub. Year	No. of methods used	Procedures including who conducted the recruitment, where it took place and what was done	Active, passive or a mixture of approaches
		outdoor banners (8 ft × 3 ft); 3 door drops (4,500 residences each drop); street leafleting; leaflet distribution via schools, community groups and GPs; a poster campaign; face to face recruitment; local press; a campaign blog; a text campaign; two taster sessions; and a loyalty scheme.	
Brady, 2010[38]	1	Men aged 40–60 were invited using the Rangers and Celtic season ticket holders' databases.	Not specified
Foster, 2013[39]	Multiple	Participants were recruited from the clinical research database of the Washington University School of Medicine (WUSM) Movement Disorders Center, the WUSM Research Participant Registry, neurologists in the St. Louis area, and advertisements in the newsletter of the Greater St. Louis Chapter American Parkinson Disease Association.	Passive
Lee, 2011[40]	3	Participants were recruited via distributed brochures, word of mouth, and local electronic newsletters.	Active/Passive
Lupton, 2003[41]	Multiple	Focus local action including social marketing through mass media, local media coverage, and partnerships	Active/Passive
Solomon, 2014[42]	Multiple	Local press (newspapers, newsletters, and radio), and with posters in local sports centres and village halls.	Passive

Table 8. Settings and location of recruitment, study and populations

Study Number, Author and Pub. Year	Stated study setting	Target population	Where did the recruitment take place?	Intervention delivery site	Where participants came from
Bryan, 2012[20]	Community	Adult men and women between 18 and 65 years who were healthy but who categorized themselves as having been physically inactive for the previous 6 months.	In the vicinity of the participating yoga studio	Community – Local yoga studio	Community
Cunningham, 1987[21]	Community/ Workplace	Men, retiring from work, while still employed (to enter the study 2 to 4 months prior to retirement).	Recruited through their company's personnel office, newspaper advertisements, and by word of mouth.	Community outdoor/ indoor track	Community/ Workplace
Murrock, 2006[22]	Community (church)	Sedentary African American women, ages 40 years and older.	During/after Sunday church service and advertised at the church.	Church	Church community
Vahabi , 2015[23]	Community	Adult South Asian immigrant women; engaged only in minimal (i.e., <1 hour per week) or no physical activity.	Community health centre	Community - Athletic Centre/ Dance Studio	Community
Wyke, 2015[24]	Community (supporters of professional sport)	Men aged 35–65 years with a BMI of at least 28 kg/m2	In football clubs and club related settings	Community – Football club home stadium	27% (n= 327) match-day recruitment men, 13% (n=154) club websites, articles or news pieces in the media and through word of mouth, 6% (n=77) work-based advertisement (6%), 5% (n=55)

Study Number, Author and Pub. Year	Stated study setting	Target population	Where did the recruitment take place?	Intervention delivery site	Where participants came from
					posters at the clubs, 4% (n=45) other club associations, 3% (n=40) fans' websites and GPs 1% (n=9).
Alexander, 2012[25]	Community	Individuals with or at risk of type 2 diabetes.	Advertising in community newspapers, university buildings, community venues, and medical offices.	Not specified	Community
Boyd, 2006[26]	Community/Care	Individuals over 19 with type 2 diabetes.	Community health centre	Community health centre	Community health centre
Jepson, 2015[27]	Care (GPs)	Severely obese adults.	GP surgeries	Clinical - Rehabilitation Centre	GP surgeries
Mangeri, 2014[28]	Care (clinical)	Adults with type II diabetes and/or obesity	From two diabetes/metabolic units	Community – local disco hall	Clinical
McDonoughn, 2010[29]	University and community	Overweight women.	University campus and in the local community	Not specified	University campus and community
Parnell, 2015[30]	Community (supporters of professional sport)	Older adults aged 55+ years	Local media, flyers, posters, club related events and promotional materials. Sometimes health centres and other older adult hubs. Word of mouth.	Community – predominantly football club facilities	Community
Pfister, 2013[31]	Workplace	Physically inactive female hospital staff members aged 45 and older.	Participants were recruited among female staff members	Not specified	Workplace

Study Number, Author and Pub. Year	Stated study setting	Target population	Where did the recruitment take place?	Intervention delivery site	Where participants came from
			of the University Hospital in Zurich, Switzerland		
Pringle, 2013[32]	Community (supporters of professional sport)	Varied between sites: typically men, 18-35 years, as well as other groups of hard-to-engage-men, including, black and minority ethnic, homeless and unemployed men.	Match days, local groups, public services, local champions, informal networks	Community - Football club facilities and community venues.	Community
Pringle, 2014[33]	Community (supporters of professional sport)	Clubs targeted men of different demographic profiles and this was reflected in the focus of the interventions. All of the interventions were directed at men aged 18-35 years, although adult men of any age were eligible to attend. 11 of the 16 interventions also targeted men over the age of 35. 15 of the 16 interventions targeted white British males. 1 intervention targeted only BME males. 8 interventions targeted white and BME males. 14 interventions targeted men in and out of employment, 2 interventions only targeted unemployed males.	A variety of locations in the local community across the different delivery sites. E.g. promotional events at clubs, local job centres. raising exercises delivered on match days to the supporters (varied across programme locations).	Community - football stadia/ club venues/ other local community venues	Community
Sport England, 2014b[34]	University	University students	Coaches, volunteers and ambassadors	Multiple	University students
Sport England, 2014c[35]	A range of settings; community, workplace and care	Inactive individuals, 14 years and above. Individual programmes may also target other factors such as medical conditions, demographic or health risk groups.	Varied across programme locations:<br>- 8 projects utilise self-referral<br>- 10 utilise referrals/ signposting from health professionals	Multiple	A range of settings; community, workplace and care

Study Number, Author and Pub. Year	Stated study setting	Target population	Where did the recruitment take place?	Intervention delivery site	Where participants came from
			- 4 utilise referrals/ signposting from other sources such as slimming clubs, community organisations etc.		
Vivian, 2014[36]	Community/Care	Women, particularly those living in deprived areas or caring for children.	A range of locations, GP practices, partner organisations	Multiple	Community
Withall, 2012[37]	Community	Individuals with an awareness of the benefits of exercise and positive attitudes towards it; anxiety-related barriers to joining exercise sessions; a fear of arriving alone (lack of relatedness); and a perceived lack of competence and autonomy.	In the community via word of mouth, leaflets, local press, taster sessions, community groups, schools and GP's.	Community - Local leisure centre	Community
Brady, 2010[38]	Community (supporters of professional sport)	Men aged 40-60 with a high BMI.	From Rangers and Celtic season ticket holders' database	Community – Football stadium	Community
Foster, 2013[39]	Care (clinical)	Adults with Parkinson disease.	Clinical research database	Not specified	Clinical research database
Lee, 2011[40]	Community	Women of colour	Brochures, word of mouth and local electronic newsletters	Not specified	Community
Lupton, 2003[41]	Community	Population of Båtsford.	Local community	Community - Multi-site	Community
Solomon, 2014[42]	Community	Residents of selected rural villages/regions of Devon, south-west England.	Local press (newspapers, newsletters, and radio), and with posters in local sports centres and village halls.	Community - primary and secondary schools, sports	Community

Study Number, Author and Pub. Year	Stated study setting	Target population	Where did the recruitment take place?	Intervention delivery site	Where participants came from
				clubs, and other partnerships	

Table 9. Recruitment rates and efficiency ratios

Study Number, Author and Pub. Year	Pool	Invited	Responded	Started	Efficiency A (%) Started/ Pool	Efficiency B (%) Started/ Invited	Efficiency C (%) Started/ Responded	Weekly recruitment rate
Bryan, 2012[20]	Not specified	Not specified	45	**Intervention** 16 **Control** 15	-	-	**Overall** 69%	3.75 / week
Cunningham, 1987[21]	Not specified	Not specified	224	**Intervention** 113 **Control** 111			**Overall** 100%	0.28 / week
Murrock, 2006[22]	Not specified	Not specified	Not specified	**Intervention** 66 **Control** 60	-	-	-	16.5 /week
Vahabi , 2015[23]	Not specified	Not specified	Not specified	27	-	-	-	3.38 / week
Wyke, 2015[24]	Not specified	Not specified	1231	**Intervention** 374 **Control** 374	-	-	**Overall** 61% **Intervention/Control** 30.5%	37.4 / week
Alexander, 2012[25]	94 (intervention and controls)	80 (intervention and controls)	Not specified	**Intervention** 39 **Control** 39	41%	49%	-	-
Boyd, 2006[26]	130	Not specified	Not specified	48	37%	-	-	-
Jepson, 2015[27]	Not specified	Not specified	53	49	-	-	92%	-
Mangeri, 2014[28]	Not specified	Not specified	Not specified	**Dance** 42	-	-	-	-

Study Number, Author and Pub. Year	Pool	Invited	Responded	Started	Efficiency A (%) Started/ Pool	Efficiency B (%) Started/ Invited	Efficiency C (%) Started/ Responded	Weekly recruitment rate
				**Self-selected PA** 58				
McDonoughn, 2010[29]	Not specified	Not specified	Not specified	**Intervention** 34 **Control** 47	-	-	-	-
Parnell, 2015[30]	Not specified	Not specified	Not specified	985	-	-	-	-
Pfister, 2013[31]	1249	275	250	68	5%	25%	27%	-
Pringle, 2013[32]	Not specified	Not specified	Not specified	Not specified	-	-	-	-
Pringle, 2014[33]	Not specified	Not specified	Not specified	4,020	-	-	-	-
Sport England, 2014b[34]	Not specified	Not specified	Not specified	160,018	-	-	-	-
Sport England, 2014c[35]	Not specified	Not specified	67,426	25,231 inactive	-	-	-	-
Vivian, 2014[36]	Not specified	Not specified	Not specified	143,774	-	-	-	-
Withall, 2012[37]	3553	Not specified	Not specified	364	10%	-	-	14.56 / week
Brady, 2010[38]	Not specified	42	40	40	-	95%	100%	-
Foster, 2013[39]	Not specified	Not specified	62	**Intervention** 26 **Control** 26	-	-	84%	-

**Study Number, Author and Pub. Year**	**Pool**	**Invited**	**Responded**	**Started**	**Efficiency A (%) Started/ Pool**	**Efficiency B (%) Started/ Invited**	**Efficiency C (%) Started/ Responded**	**Weekly recruitment rate**
Lee, 2011[40]	Not specified	Not specified	95	**Intervention** 25 **Control** 25	-	-	53%	-
Lupton, 2003[41]	2,500	**Intervention** 595 **Control** 1362 invited to the survey	**Intervention** 364 **Control** 960 completed the survey	**Intervention** 186 **Control** 482	**Overall** 27% **Intervention** 7% **Control** 19%	**Overall** 34% Intervention 31% **Control** 35%	**Overall** 50% Intervention 51% **Control** 50%	-
Solomon, 2014[42]	Not specified	32,315 invited to complete the evaluation survey	10,412 completed the postal survey (32%)	Not specified	4% of randomly surveyed villagers reported participating	-	-	-

Table 10. Physical activity outcomes (in intervention participants if controlled trial, significant differences compared to control where applicable)

Study Number, Author and Pub. Year	N providing baseline PA	N followed up at end of intervention	N followed up post-intervention	Measure and units of PA used	PA outcome change at end of intervention	PA outcome change at post-intervention follow-up
Bryan, 2012[20]	16	15 (10 weeks)	15 (15 weeks)	7-day Physical Activity Recall, Hours/week	↑PA $p<0.05$	No significant difference in PA
Cunningham, 1987[21]	113	100 (12 months)	-	Minnesota Leisure Time Activity questionnaire (Mets/week)	↑ high intensity PA $p<0.05$	-
Murrock, 2006[22]	66	46 (8 weeks)	46 (18 week)	Physical Activity Scale for the Elderly (PASE score)	↑PA $p<0.05$	No significant difference in PA
Vahabi , 2015[23]	27	27 (6 weeks)	-	Attendance register	Average attendance was 85% (range, 77%-96%). All participants indicated that the dance program had a variety of important physical, mental, and social health benefits	-
Wyke, 2015[24]	374	330 (12 weeks)	333 (12 months)	International Physical Activity Questionnaire (IPAQ, Short Form) (MET-min/week) for walking, vigorous, and moderate exercise.	↑PA $p<0.0001$	No significant difference in PA
Alexander, 2012[25]	39	33 (3 months post)	32 (6 months post) 36	Physical Activity Scale for the Elderly (PASE score)	No significant difference in PA	No significant difference in PA

Study Number, Author and Pub. Year	N providing baseline PA	N followed up at end of intervention	N followed up post-intervention	Measure and units of PA used	PA outcome change at end of intervention	PA outcome change at post-intervention follow-up
			(15 months post)			
Boyd, 2006[26]	130	48<br>(12 months)	-	Attendance/ Membership usage	**Exercise Categories**<br>56.3% “experimental exercisers” (1-11 visits/year)<br>25% “involved exercisers.” (12-47 visits/year)<br>19% “regular exerciser” (≥48 visits/year)	-
Jepson, 2015[27]	49	48<br>(6 weeks)	38<br>(12 months)<br>27<br>(24 months)	Actigraph accelerometer, (counts per minute)	↑PA $p<0.05$	↑PA $p<0.05$<br>(12 months)<br>No significant difference in PA<br>(24 months)
Mangeri, 2014[28]	**Dance**<br>42<br>**Self-selected PA**<br>58	**Dance**<br>41<br>**Self-selected PA**<br>54<br>(3 months)	**Dance**<br>41<br>**Self-selected PA**<br>52<br>(6 months)	Attendance and self-reported PA in PA diary used to calculate energy expenditure (MET-hour per week)	**Dance** ↑PA $p<0.05$<br>**Self-selected PA**<br>No significant difference in PA	**Dance** ↑PA $p<0.05$<br>**Self-selected PA**<br>No significant difference in PA
McDonoughn, 2010[29]	34	33	33<br>(6 months)	Self-report physical activity i.e. types and amount of activity used to calculate energy expenditure (kCal/week)	↑PA $p<0.05$	No significant difference in PA
Parnell, 2015[30]	985	486<br>(12 weeks)	-	Not specified	As the frequency of PA increased participants	-

Study Number, Author and Pub. Year	N providing baseline PA	N followed up at end of intervention	N followed up post-intervention	Measure and units of PA used	PA outcome change at end of intervention	PA outcome change at post-intervention follow-up
					reported feeling healthier and fitter	
Pfister, 2013[31]	68	47 (3 months)	46 (12 months)	Freiburger Physical Activity Questionnaire was used to calculate energy expenditure (kCal/week)	↑PA $p<0.0001$	↑PA $p<0.0001$
Pringle, 2013[32]	QUAL	QUA	QUAL	Qualitative	↑PA $p<0.001$	Qualitative
Pringle, 2014[33]	4020	2917 (12 weeks)	-	Activity recorded as sessions/week, (no. of days of ≥30mins of moderate intensity PA)	↑PA $p<0.0001$	-
Sport England, 2014b[34]	160,018	-	-	Sport PA question (sport mins/week)	2% increase in once a week sport participation	2% increase maintained into year 3
Sport England, 2014c[35]	25,231	>9,639 (end of intervention follow-up)	>979 (3 months post)	Sport PA question (sport mins/week)	9,639 inactive people doing >1 x 30 minutes of activity through sport/week	979 people >1 x 30 minutes of activity through sport
Vivian, 2014[36]	143,774	989 (~12 months)	528 (~24 months)	Self-report change in PA (descriptive) (increased, decreased, stayed the same)	77% of participants ↑PA	38% of participants ↑PA
Withall, 2012[37]	364	-	-	Attendance (sessions/ month)	Intervention sessions had significantly higher monthly attendance	-

Study Number, Author and Pub. Year	N providing baseline PA	N followed up at end of intervention	N followed up post-intervention	Measure and units of PA used	PA outcome change at end of intervention	PA outcome change at post-intervention follow-up
					rates than either the pre-existing or control area sessions, in all 6 months of the intervention p<0.01	
Brady, 2010[38]	40	40	-	Attendance/ retention	100% attendance, i.e. ≥60 minutes of PA/week for 10 weeks	-
Foster, 2013[39]	36	26 (3 months)	16 (6 and 12 months)	Activity Card Sort (ACS) (participation in daily life activities)	↑PA p<0.05	↑PA p<0.05
Lee, 2011[40]	50	43 (4 weeks)	41 (8 weeks) 36 (12 weeks)	Godin Leisure-Time Exercise Questionnaire (Met-min/week and kCal) Actigraph (≥moderate PA minutes)	↑PA P<0 .001	↑PA P<0 .001
Lupton, 2003[41]	364	Repeated cohort survey 364 (6 years)	-	Classified as active if achieving a minimum of four hours of weekly moderate physical activity during the last year	↑PA p<0.05 (>4hrs/week) in males only, no significant difference in females or the whole participant population	-
Solomon, 2014[42]	Baseline postal survey – control group only	4% of those completing intervention	Repeated cross-sectional Postal survey	International physical activity questionnaire	No significant increase in odds of adults meeting PA guidelines	-

Study Number, Author and Pub. Year	N providing baseline PA	N followed up at end of intervention	N followed up post-intervention	Measure and units of PA used	PA outcome change at end of intervention	PA outcome change at post-intervention follow-up
		location questionnaires participated (n~183)	4,584	(IPAQ) short-form (meeting PA guidelines; Minutes spent in moderate-and-vigorous activity per week)	in intervention villages p>0.05 (adjusted OR 1.02, 95% CI: 0.88 to 1.17; P = 0.80). Non-significant increase in minutes of moderate-and-vigorous-intensity activity per week (adjusted mean difference = 171, 95% CI: -16 to 358; P = 0.07).	

Table 11. Recruitment target, exclusion and study retention

Study Number, Author and Pub. Year	Sample size calculation (Yes/No) percentage achievement of sample size	Eligibility screening (Yes/No) proportion excluded	None-use attrition rates (i.e. none attenders)	Cost of recruitment per participant	Use of recruitment incentives (Yes/No), what?	Attendance average (%)	Retention rate	Number and reason for dropouts	Incentives or interventions to improve retention (Yes/No), what?
Bryan, 2012[20]	Not specified	Yes - 11% ineligible from screening	Not specified	Not specified	Not specified	75% (12/16)	Not specified	Not specified	Not specified
Cunningham, 1987[21]	Not specified	Not specified	Not specified	Not specified	Not specified	**Workout frequency** **0-3 months** 2.6±0.9 sessions/week **3-6 months** 2.5±0.9 sessions/week **6-9 months** 2.3±0.9 sessions/week **9-12 months** 2.4±0.9 sessions/week	Completed follow-up 80% intervention group	20% (23/113) of the activity group participants were classified as dropouts. Reasons: moved away (n=1), experienced illness or injury (n=6), returned to work (n=6), refused to continue (n=10).	Not specified
Murrock, 2006[22]	The study was insufficiently	Yes – 2% ineligible	8% (5/66)	Not specified	Not specified	22.5% attended 1-6 dance classes,	Completed follow-up	30% (20/66) dropped out of the experimental	Not specified

Study Number, Author and Pub. Year	Sample size calculation (Yes/No) percentage achievement of sample size	Eligibility screening (Yes/No) proportion excluded	None-use attrition rates (i.e. none attenders)	Cost of recruitment per participant	Use of recruitment incentives (Yes/No), what?	Attendance average (%)	Retention rate	Number and reason for dropouts	Incentives or interventions to improve retention (Yes/No), what?
	powered. The target recruitment figure was 78 women in each arm. 81% of the target recruitment figure was achieved.	from screening				22.5% attended 7-11 dance classes, 47% attended 12-16 dance classes	70% intervention group	group. The majority of these women had health concerns or pressing family issues, some had changes in their work schedule, and three were lost to follow up.	
Vahabi , 2015[23]	Not specified	Yes -. Not specified	0%	Not specified	Not specified	85% (10/12)	Not specified	10% (3/30) of women dropped out due to illness, unexpected travel abroad and other unexpected commitments that came up.	Not specified
Wyke, 2015[24]	Study was sufficiently powered. The target recruitment figure was 360 men in	Yes – 6% ineligible from screening	Not specified	Not specified	Not specified	73% (8.7/12)	Completed follow-up Intervention group **12 weeks** 88% **1 year**	46 participants reported reasons for dropout. 41% due to changes to work patterns	£40 club voucher for completing follow-up measures

Study Number, Author and Pub. Year	Sample size calculation (Yes/No) percentage achievement of sample size	Eligibility screening (Yes/No) proportion excluded	None-use attrition rates (i.e. none attenders)	Cost of recruitment per participant	Use of recruitment incentives (Yes/No), what?	Attendance average (%)	Retention rate	Number and reason for dropouts	Incentives or interventions to improve retention (Yes/No), what?
	each arm. 103% of the target recruitment figure was achieved.						89%	(n=19), 15% (n=7) injured, 11% (n=5) health, 2% (n=1) did not like the classroom sessions, 4% (n=2) dislike the PA sessions. 35% (n=16) 'other reasons'.	
Alexander, 2012[25]	Not specified	Yes - Not specified	Not specified	Not specified	Not specified	Not specified	Completed follow-up **3 months** 74% **6 months** 75% **15 months** 81%	15% (14/94) didn't consent to follow-up. Reasons for attrition included schedule conflicts/competing obligations (n = 3), health problems (n = 4), abnormal laboratory values (n = 2), distance (n= 1), or unknown reasons (n = 4).	Not specified

Study Number, Author and Pub. Year	Sample size calculation (Yes/No) percentage achievement of sample size	Eligibility screening (Yes/No) proportion excluded	None-use attrition rates (i.e. none attenders)	Cost of recruitment per participant	Use of recruitment incentives (Yes/No), what?	Attendance average (%)	Retention rate	Number and reason for dropouts	Incentives or interventions to improve retention (Yes/No), what?
Boyd, 2006[26]	Not specified	Yes - Not specified	63% (82/130)	Not specified	Not specified	The majority (56%) of participants were categorised as "experimental exercisers" attending 1-11 classes.	19% of participants were categorised as "regular exercisers" averaging 7.6 visits per month.	Not specified	Free 3 month membership and a reduced membership rate afterwards.
Jepson, 2015[27]	Calculation of sample size and power was done. This study has 71 to 73 observations for the main outcomes. Study was sufficiently powered but not stated whether this was a target	Yes - Not specified	Not specified	Not specified	Not specified	Not specified	Completed follow-up **6 weeks** 98% **1 year** 78% **2 years** 55%	**2 years** 45% (22/49) lost to follow-up. Reasons for dropout: 23% (n=5) problems with the study protocol, 77% (n=17) referral to obesity surgery, pregnancy, reaching personal weight goals, health problems, inability to attend the residential	Not specified

Study Number, Author and Pub. Year	Sample size calculation (Yes/No) percentage achievement of sample size	Eligibility screening (Yes/No) proportion excluded	None-use attrition rates (i.e. none attenders)	Cost of recruitment per participant	Use of recruitment incentives (Yes/No), what?	Attendance average (%)	Retention rate	Number and reason for dropouts	Incentives or interventions to improve retention (Yes/No), what?
	recruitment figure							stays, or unknown reasons.	
Mangeri, 2014[28]	Not specified	Yes - Not specified	Not specified	Not specified	Not specified	**Dance**<br>83% (34/41) attending >70% of planned sessions.	Completed follow-up<br>**3 months**<br>**Dance**<br>98%<br>**Self-selected activity**<br>93%	Not specified	Not specified
McDonoughn, 2010[29]	Not specified	Yes - Not specified	19% (15/81)	Not specified	Not specified	18% (n=6) of the experimental group attended fewer than 10 sessions	End of intervention 100%	0	Not specified
Parnell, 2015[30]	Not specified	Not specified	Not specified	Not specified	Not specified	Not specified	Completed follow-up 49%	Not specified	Not specified
Pfister, 2013[31]	Not specified	Yes - 46% ineligible from screening	Not specified	Not specified	Not specified	80%	Completed the whole programme 69%	31% (21/68) dropped out for the following reasons: poor health (n = 12),	Not specified

Study Number, Author and Pub. Year	Sample size calculation (Yes/No) percentage achievement of sample size	Eligibility screening (Yes/No) proportion excluded	None-use attrition rates (i.e. none attenders)	Cost of recruitment per participant	Use of recruitment incentives (Yes/No), what?	Attendance average (%)	Retention rate	Number and reason for dropouts	Incentives or interventions to improve retention (Yes/No), what?
								lack of time (n = 6), conflicting job (n = 1) and unknown reason (n = 2)	
Pringle, 2013[32]	Not specified	Not specified	Not specified	Not specified	Not specified	Not specified	Not specified	Not specified	Not specified
Pringle, 2014[33]	Not specified	Not specified	Not specified	Not specified	Not specified	Not specified	Completed follow-up 73%	Not specified	Not specified
Sport England, 2014b[34]	Not specified	Not specified	Not specified	Not specified	Not specified	Average of 10 visits per participant	Not specified	Not specified	Not specified
Sport England, 2014c[35]	Not specified	Yes - Not specified	Not specified	Not specified	Not specified	Not specified	End of intervention 38% inactive people achieving >1 x 30 minutes of activity through sport	Not specified	Not specified

Study Number, Author and Pub. Year	Sample size calculation (Yes/No) percentage achievement of sample size	Eligibility screening (Yes/No) proportion excluded	None-use attrition rates (i.e. none attenders)	Cost of recruitment per participant	Use of recruitment incentives (Yes/No), what?	Attendance average (%)	Retention rate	Number and reason for dropouts	Incentives or interventions to improve retention (Yes/No), what?
Vivian, 2014[36]	Not specified	Varied by local programme	Not specified	Not specified	Not specified	**A**verage attendances per participant **Year One** 3.9 **Year Two** 3.8 **Year Three** 4.8	Not specified	Not specified	Not specified
Withall, 2012[37]	Not specified	Not specified	Not specified	£20.32 (7395/364)	Not specified	364 individuals attended at least one session over the six month intervention	Intervention session attendance was significantly greater in all six months than at pre-existing and control area sessions; **Month 1** 38.83 (p<0.001)	The intervention's drop-out rate was similar to that in the control area (66.2% v 69.9%), and considerably lower than in the pre-existing sessions (83%).	Not specified

Study Number, Author and Pub. Year	Sample size calculation (Yes/No) percentage achievement of sample size	Eligibility screening (Yes/No) proportion excluded	None-use attrition rates (i.e. none attenders)	Cost of recruitment per participant	Use of recruitment incentives (Yes/No), what?	Attendance average (%)	Retention rate	Number and reason for dropouts	Incentives or interventions to improve retention (Yes/No), what?
							**Month 2** 21.45 (p<0.001) **Month 3** 9.57 (p<0.001) **Month 4** 17.35 (p<0.001) **Month 5** 20.33 (p<0.001) **Month 6** 28.72 (p<0.001)		
Brady, 2010[38]	Not specified	Yes - Not specified	5% (2/40)	Not specified	Not specified	100%	Completed follow-up 100%	There were two early withdrawals prior to the initial assessment, and these places were readily filled by others.	Not specified
Foster, 2013[39]	Not specified	Yes - Not specified	19% (6/32)	Not specified	Not specified	80%	81% of intervention group received	Lost to follow-up/discontinued intervention (n=10), unrelated	Not specified

Study Number, Author and Pub. Year	Sample size calculation (Yes/No) percentage achievement of sample size	Eligibility screening (Yes/No) proportion excluded	None-use attrition rates (i.e. none attenders)	Cost of recruitment per participant	Use of recruitment incentives (Yes/No), what?	Attendance average (%)	Retention rate	Number and reason for dropouts	Incentives or interventions to improve retention (Yes/No), what?
							the intervention	medical issues (n=6), scheduling conflict (n=1), stopped attending (n=1), left the country (n=1), deep brain stimulation surgery (n=1)	
Lee, 2011[40]	Not specified	Yes – 25% ineligible from screening	Not specified	Not specified	Not specified	**Group 1** 63% **Group 2** 57%	Not specified	Not specified	Not specified
Lupton, 2003[41]	Study was sufficiently powered but not stated whether this was a target recruitment figure.	Not specified	Not specified	Not specified	Not specified	Not specified	From 1987 to 1993, the increase in the proportion of males who were physically active in Ba° tsfjord was 8.6% while the	Not specified	Not specified

Study Number, Author and Pub. Year	Sample size calculation (Yes/No) percentage achievement of sample size	Eligibility screening (Yes/No) proportion excluded	None-use attrition rates (i.e. none attenders)	Cost of recruitment per participant	Use of recruitment incentives (Yes/No), what?	Attendance average (%)	Retention rate	Number and reason for dropouts	Incentives or interventions to improve retention (Yes/No), what?
							increase in the control community was 0.6% (pv0.047)		
Solomon, 2014[42]	Study was sufficiently powered but not stated whether this was a target recruitment figure	Not specified	Not specified	Not specified	Not specified	Not specified	Not specified	Not specified	Not specified

Table 12. Additional comments regarding recruitment and retention

**Study Number, Author and Pub. Year**	**Comments/evaluation data regarding recruitment in the write up, protocol or allied papers**	**Comments about retention in the write up, protocol or allied papers**
Bryan, 2012[20]	-	-
Cunningham, 1987[21]	-	-
Murrock, 2006[22]	-	-
Vahabi , 2015[23]	-	- There was a high participation rate.<br>- Participants demonstrated very high overall satisfaction with the programme, which is an important motivator to participation in exercise. This could be explained by the programme characteristics, specifically that it was culture and gender specific, and tailored to the target group of South Asian women.<br>- The social environment provided in this study was an important factor in encouraging women's participation in physical activity. Many participants highlighted the benefit of the fun environment where they interacted with other women and were able to concentrate on themselves.
Wyke, 2015[24]	- Those attracted to the programme were already at least partly motivated to make changes to the lifestyles given the right opportunity.<br>- The intervention may not attract those who have not already considered weight loss.<br>- The intervention is also unlikely to attract those who are actively disinterested in football or other sports and other approaches will be necessary.	Examination of participant attendance week by week over the 12-week programme demonstrated that there was an initial drop in attendance between sessions 1 and 5, after which attendance remained relatively stable until the final two sessions, when a further small drop-off was observed.
Alexander, 2012[25]	-	-

Study Number, Author and Pub. Year	Comments/evaluation data regarding recruitment in the write up, protocol or allied papers	Comments about retention in the write up, protocol or allied papers
Boyd, 2006[26]	-	The programme's ability to directly coach the patient in a controlled environment and to provide long-term motivation was thought to have contributed to the higher exercise adherence.
Jepson, 2015[27]	-	-
Mangeri, 2014[28]	-	All studies involving gyms need an intense support to limit attrition, whereas the social support of dancing was probably pivotal to keep patients with diabetes on treatment.
McDonoughn, 2010[29]	-	-
Parnell, 2015[30]	- Many participants reported that the most attractive aspect of the intervention was the regular opportunity for social support and interaction; this included the opportunity to meet new people.<br>- Participants reported that the 'football club's badge' helped to reach older adults and had a positive influence on participants' interest in the programme.<br>- Successful recruitment often involved getting 'buy in' from the participants and attracting those who had older adult caring responsibilities.<br>- One of the most powerful ways of connecting with potential participants was through word of mouth; the social advantages being advocated in these exchanges were important outreach approaches.<br>- Typically, 'word-of-mouth' provided reassurance to potential participants about returning to PA; these recommendations were powerful in allaying apprehensions about attending an unfamiliar programme. Receiving a recommendation from 'someone like me' makes a big difference compared to a similar endorsement from a stranger or someone much younger.	Participants also reported that they enjoyed being active through a varied programme of social and physical activities. These activities were offered routinely and for a number of participants weekly routine was central to their attendance.

Study Number, Author and Pub. Year	Comments/evaluation data regarding recruitment in the write up, protocol or allied papers	Comments about retention in the write up, protocol or allied papers
	- Linking with key community workers were helpful for recruiting potential participants who were unlikely to encounter promotional events.<br>- Intervention staff aspired to engage with a range of service providers.<br>- For a number of individuals playing football was important.<br>- Providing a flexible menu of activities and timings was considered an important design characteristic.<br>- Consultation with older adults when designing the intervention appears to have contributed to strong recruitment and impressive adherence	
Pfister, 2013[31]	-	- It is hypothesised that participants knew each other before the programme, wanted to join the programme together and motivated each other during the programme which resulted in the sustained benefit due to a group effect even beyond the end of the intervention.<br>- A higher baseline self-motivation towards physical activity was associated with increased likelihood of participating in the offered exercise programme ($P < 0.001$). Only 13% of the interested women with low motivation started the programme with 24% moderately motivated women and up to 40% highly motivated women.<br>- Adherence to the programme also appeared to be associated with motivation level with borderline statistical significance ($P = 0.05$). The proportion of adherent women was the highest (77%) among women in the high motivation level group, 70% in the moderately motivated women and 33% in the women with low motivation.

Study Number, Author and Pub. Year	Comments/evaluation data regarding recruitment in the write up, protocol or allied papers	Comments about retention in the write up, protocol or allied papers
Pringle, 2013[32]	- The recruitment successfully reached men demonstrating multiple health behaviours which contribute to chronic conditions, but also men who did not report having a health problem.<br>- Engaging men typically regarded as 'hard to contact or engage' (i.e. over one-third of men who reported that they did not consult their GP, and over half of men who never engaged with a health advice service such as NHS Direct).<br>- Unemployed men and men who reported no or limited social support networks were also recruited. This further underlines the value of male-specific interventions, since the aforementioned groups can otherwise be characterized as 'at risk' in health terms.<br>- Despite having substantial health needs these participants were unlikely to be exposed to conventional health promotion opportunities made available through these channels.<br>- Health interventions delivered in professional football clubs have a powerful reach with male supporters, but also with men not engaging with primary care and health information services.<br>- Just under one-third of the participants were not fans of the host club. This result suggests that factors other than the draw of the football club may be influential in facilitating the engagement of 'non-fans'.<br>- Focused efforts are required to engage these groups of men into health-enhancing activities. For instance, in this study, two of the interventions recruited men from local job centres.	-
Pringle, 2014[33]	- This study has shown the importance of interventions being viewed by men as 'acceptable.'<br>- Over 85% of men in this study presented with multiple combinations of CVD risk factors, yet a similar percentage did not see themselves as 'having health problems and over half viewed their health as at least "good"'.	Men reported that their participation was helped by specific design factors:<br>- Regularity of weekly classes and groups.<br>- Delivering the interventions in nearby, convenient and/or familiar football/community venues.

Study Number, Author and Pub. Year	Comments/evaluation data regarding recruitment in the write up, protocol or allied papers	Comments about retention in the write up, protocol or allied papers
	- Men aged 18–44 dominated engagement rates. Thus comparing favourably with recruitment levels found at community physical activity interventions, where this group of men have been underrepresented.<br>- Black and minority ethnic group (BME) men made up one-third of the overall sample.<br>- Word of mouth helped facilitate men's adoption rates, including that of friends.<br>- Engagement of 'non-club supporters' demonstrated that the football/sporting environment was appealing to the target group regardless of an interest in premier league football or the delivering club in particular.<br>- Football and the football club provided a powerful hook for reaching men. Club infrastructure (match-day events, players, ground, badge) was also deemed important for reaching fans with information on the intervention.	- Activities were populated by like-minded men, providing regular opportunities for social engagement.<br>- Social interaction was a commonly reported benefit.<br>- Opportunities to develop supportive relationships,<br>- Enjoying 'being active' along with achievement, fitness and fun, among the most commonly discussed themes when talking about programme outcomes.<br><br>Men did not find all elements of the interventions acceptable. For instance:<br>- While competition can appeal to men, it may also be a barrier to some,<br>- Similarly, group physical activity can also be daunting initially. Participants therefore require a gradual and supportive induction to interventions, particularly in apprehensive men.<br>- A broad programme of physical activity options, in addition to football, is needed to cater for diverse needs.
Sport England, 2014b[34]	- Word of mouth communication with peer encouragement, worked most effectively with students.<br>- Social media, emails and printed posters were also rated highly by projects. Promotional and viral videos were also mentioned, as were pop-up stalls and taster opportunities in high footfall locations.<br>- Projects also highlighted the importance of choosing wording and imagery carefully in order to create the right impression for the target group.<br>- Posters/fliers on their own were said to have limited impact, according to feedback from participants, but backed up by talking to individuals/groups they serve to confirm and remind people as they walk around campus.	- Students' interest and readiness to participate can vary over time, so maintaining an agile and flexible offer helps to ensure that they can participate when they want to.<br>- Some Universities found that having leagues can help retention in some sports, as students feel obliged to find someone to take their place if they can't attend a session, and many of the replacements go on to become regular participants themselves.

Study Number, Author and Pub. Year	Comments/evaluation data regarding recruitment in the write up, protocol or allied papers	Comments about retention in the write up, protocol or allied papers
	- Sessions that are marketed as fitness classes will attract 'non-sporty' participants who are looking for a session. I.e. those who haven't been confident enough to turn up to a 'basic' sports coaching session, but are more comfortable with attending a fitness session.	
Sport England, 2014c[35]	**Community Engagement**<br>- The presence of Activators, Mentors and Community Champions in communities appears to be key to gaining the trust of communities.<br>- Embedding projects within other community activities can be a critical recruitment tool, for instance, working with faith groups to develop sporting activities for their members.<br>- Tailoring of marketing and the "sport" offer is key.<br>- Use of the term sport may be off-putting for the inactive target group. Projects have had to develop different ways of thinking to market their activities effectively to this group.<br>- Taster sessions are a useful recruitment tool for projects, giving people the opportunity to come and try activities before signing up for greater involvement.<br>- Mass mail out techniques have been effective at engaging and recruiting inactive people.<br>- Online sign up systems can be efficient and effective approaches to engage with inactive people, particularly when projects are multi-site in their delivery.<br>- Automatic communication triggers can be a useful tool for engagement.<br>- Social Media and Word of Mouth are key recruitment tools. >59% of recruitment for some projects has been through word of mouth with >16% hearing about the projects through social media.<br>- Refining recruitment systems can see increases in the number of inactive people engaging with project of >15%.	-

Study Number, Author and Pub. Year	Comments/evaluation data regarding recruitment in the write up, protocol or allied papers	Comments about retention in the write up, protocol or allied papers
	- Social bonding approaches, such as using community coffee mornings to engage inactive people and enable them to develop confidence in attending venues and in meeting new people, can be a useful precursor to sporting activities.<br>- Family and Friend based Motivational Interviewing is proving useful in enhancing success and boosting recruitment.<br>- Providing feedback to Health professionals is a key part of the referral process.	
Vivian, 2014[36]	The key activities that contributed to recruitment were:<br>- Increasingly tailored promotion, in particular an emphasis on - the social / fun aspect of the active women sessions and using images of ‘real women’.<br>- utilising ‘ambassadors’ / ‘champions’ where possible<br>- Promotion throughout the entire community, not just in sporting environments<br>- increased online promotion, social media<br>- Face-to-face recruitment / seeing sessions ‘in action’<br>- Partnership working with other organisations<br>- Providing easy-to-access practical information on sessions<br>- “active” engagement required alongside promotional materials to recruit those “hard-to-reach” women.<br>- word of mouth among women in the community remains one of the most effective recruitment channels, particularly among the harder-to-reach target group of those living in deprived areas.<br>- Finding existing groups of women and recruiting them as a group meant the women were more likely not only to turn up to the first session, but also to keep going.<br>Recruitment-related challenges include:<br>- Recruiting from target groups:	Two-fifths (41%) of participants took up another sport outside of Active Women as a result of attending the sessions. There was also evidence to suggest that participants were encouraging their friends and family to engage in more activity as well. The following measures were successfully used by projects over the course of the Active Women Programme to retain participants:<br>- Reviewing the timing of sessions on a frequent basis:<br>- Ensuring classes were low cost and affordable:<br>- Strengthening communications from project staff<br>- Tailoring sessions to fit the needs and profile of participants<br>- Ensuring staff had the right soft skills and improving the staff-to-participant ratio<br>- Offering incentives to participants<br>- Offering progression opportunities<br>- Offering a wider variety of sessions/classes.

Study Number, Author and Pub. Year	Comments/evaluation data regarding recruitment in the write up, protocol or allied papers	Comments about retention in the write up, protocol or allied papers
	- Projects found it more difficult to recruit women from deprived areas and those caring for children (the target groups), rather than women more generally.<br>- Partly due to insufficient resource (or a reluctance to deploy resources from other activities) for the face-to-face recruitment that seems to be successful for this group<br>- In the early days of the programme when participant volumes were lower some projects had successfully recruited women via active engagement. However this approach tended not to be scalable as participant volumes increased.<br>- It's possible that projects could have done more to maximise this word-of-mouth recruitment channel.<br>- Important for promotional materials to be visible in as wide a variety of settings throughout the community as possible. This was particularly resource-intensive exercise and as a result was not distributed as widely as would have been ideal.	
Withall, 2012[37]	- Direct comparisons with other approaches were difficult due to a lack of standard definitions of recruitment and adherence and little detailed reporting of findings. In this study recruitment reflects whether the target group overcame any pertinent barriers and were sufficiently motivated to engage with the intervention at least once.<br>- In months one and two, traditional marketing techniques (posters/outdoor banners/flyers) had the greatest influence on recruitment compared to word of mouth communication (84.5%v15.5%).<br>- In months five and six word of mouth influenced 57.5% of new recruits.<br>- The substantial increase in the influence of word of mouth on recruitment between the early and late phases of the intervention, indicates recruitment could have been boosted due to increased	- Attendance is the number of participants at any one session, and indicates the scale of the impact of the intervention.<br>- If participants attended for more than one month they became much more likely to adhere. The intervention's initial six week period of free sessions supported the development of a habit to increase the likelihood of long term adherence.<br>- Of all the factors affecting motivation to exercise, enjoyment was the only one which differed between adherers and non-adherers.<br>- While it is widely accepted that enjoyment is associated with exercise adherence, policy makers should consider that in order to activate output word of mouth, the most influential marketing tool, levels of enjoyment need to be very, not just moderately, high.

Study Number, Author and Pub. Year	Comments/evaluation data regarding recruitment in the write up, protocol or allied papers	Comments about retention in the write up, protocol or allied papers
	promotion via this channel. The relatively large numbers of participants increased the social networks available as communications channels, and so increased the likelihood of non-participants hearing about the intervention from multiple sources. This helped in creating a sense of a socially acceptable norm.<br>- Investment in promotion, sufficient that the target market is exposed multiple times to good quality materials and well-designed messages, is important if high levels of recruitment are to be achieved, and therefore good levels of word of mouth generated.<br>- An intervention may require a certain longevity in order to fully capitalize on the sales impact of word of mouth.<br>- New or novel forms of activity may generate interest amongst those who had previously dismissed participation in commonly available activities.	- Good attendance levels increase the capacity and reach of the word of mouth communication channel, offer evidence of peer and social support for the activity and increase the opportunities for socialising and therefore building relatedness.
Brady, 2010[38]	-	-
Foster, 2013[39]	-	- The social interaction, social support and social influences that emerged from the tango classes likely had positive effects on participation.<br>- The group setting provided an opportunity for social modelling, the establishment and reinforcement of social norms regarding health-promoting behaviour, and the development of social networks. In fact, participants in the Tango group reported engaging in social activities together outside of class, including attending a play, the symphony, and a social dance.
Lee, 2011[40]	-	-
Lupton, 2003[41]	-	-

Study Number, Author and Pub. Year	Comments/evaluation data regarding recruitment in the write up, protocol or allied papers	Comments about retention in the write up, protocol or allied papers
Solomon, 2014[42]	- Ensuring sufficient penetration and reach across a community to attain a population-level impact is one of the most difficult aspects of community-level interventions.<br>- Of the study participants in the intervention mode 16% reported awareness of Devon Active Villages, and 4% reported participation in intervention events.<br>- It is possible that the intervention was effective at the individual level, but the low levels of population penetration prevented any observable effect at the village level.<br>- There was only a limited budget for promotion activities, which may have contributed to the low levels of participant awareness in the research study.<br>- In rural areas with an ageing population, it is arguably more difficult to find effective ways of communicating new physical activity opportunities to sedentary individuals, because most methods rely on participants seeing an advertisement in the local area.<br>- Media activities (e.g., television, radio) can achieve greater levels of reach, but can also be expensive for localised community-based interventions.<br>- From the population penetration rates achieved it is clear that the intervention would be classed as ‘low reach’. Therefore, the results of the present investigation are in line with previous research, where interventions with low reach failed to have an effect on physical activity behaviour.	-
