# Supplementary material for: Recruiting adult participants to physical activity intervention studies using sport: a systematic review
Source: BMJ Open Sport Exerc Med. 2017 Jul 11;3(1):e000231. doi: 10.1136/bmjsem-2017-000231 (PMC5530105; doi:10.1136/bmjsem-2017-000231)
Supplement: Supplementary data [file bmjsem-2017-000231supp002.pdf]

SUPPLEMENTARY INFORMATION

Appendix (i) Search syntax for electronic databases

Search ID	**Search Terms**
1	intervention
2	(MH "Sports")
3	aikido
4	air sports
5	american football
6	angling
7	archery
8	arm wrestling
9	athletics
10	australian rules football
11	badminton
12	baseball
13	basketball
14	baton twirling
15	biathlon
16	billiards OR snooker
17	bobsleigh
18	boccia
19	bowls
20	boxing
21	camogie
22	canoeing
23	caving
24	chinese martial arts
25	cricket
26	croquet
27	curling
28	cycling
29	darts
30	disability sport
31	dodgeball
32	dragon boat racing
33	equestrian
34	(MH "Exercise")
35	fitness

36	dance
37	fencing
38	racket OR racquet
39	floorball
40	football
41	gaelic games
42	golf
43	gymnastics
44	handball
45	highland games
46	hockey
47	hovering
48	ice hockey
49	ice skating
50	judo
51	ju jitsu
52	kabbadi
53	karate
54	kendo
55	kite surfing
56	korfball
57	lacrosse
58	life saving
59	luge
60	pentathlon
61	motor cycling
62	motorsports OR motor sports
63	mountaineering
64	netball
65	orienteering
66	petanque
67	polo
68	pool
69	quoits
70	rambling
71	real tennis
72	roller sports
73	rounders
74	rowing
75	rugby

76	sailing
77	yachting
78	shinty
79	shooting
80	skateboarding
81	skipping
82	snowsport OR skiing OR snowboarding
83	softball
84	sombo
85	squash
86	stoolball
87	sub aqua
88	surfing
89	surf life saving
90	swimming
91	table tennis
92	taekwondo
93	tang soo do
94	bowling
95	tennis
96	triathlon
97	tug of war
98	ultimate frisbee
99	volleyball
100	water skiing
101	weight lifting OR weightlifting
102	wrestling
103	yoga
104	recruit*
105	participat*
106	market*
107	2 OR 3 OR 4 OR 5 OR 6 OR 7 OR 8 OR 9 OR 10 OR 11 OR 12 OR 13 OR 14 OR 15 OR 16 OR 17 OR 18 OR 19 OR 20 OR 21 OR 22 OR 23 OR 24 OR 25 OR 26 OR 27 OR 28 OR 29 OR 30 OR 31 OR 32 OR 33 OR 34 OR 35 OR 36 OR 37 OR 38 OR 39 OR 40 OR 41 OR 42 OR 43 OR 44 OR 45 OR 46 OR 47 OR 48 OR 49 OR 50 OR 51 OR 52 OR 53 OR 54 OR 55 OR 56 OR 57 OR 58 OR 59 OR 60 OR 61 OR 62 OR 63 OR 64 OR 65 OR 66 OR 67 OR 68 OR 69 OR 70 OR 71 OR 72 OR 73 OR 74 OR 75 OR 76 OR 78 OR 79 OR 80 OR 81 OR 82 OR 83 OR 84 OR 85 OR 86 OR 87 OR 88 OR 89 OR 90 OR 91 OR 92 OR 93 OR 94 OR 95 OR 96 OR 97 OR 98 OR 99 OR 100 OR 101 OR 102 OR 103
108	104 OR 105 OR 106
109	1 AND 107 AND 108 limited to English language and adults (19+ years)
