# Supplementary figures and images for: Recruiting adult participants to physical activity intervention studies using sport: a systematic review
Source: BMJ Open Sport Exerc Med. 2017 Jul 11;3(1):e000231. doi: 10.1136/bmjsem-2017-000231 (PMC5530105; doi:10.1136/bmjsem-2017-000231)

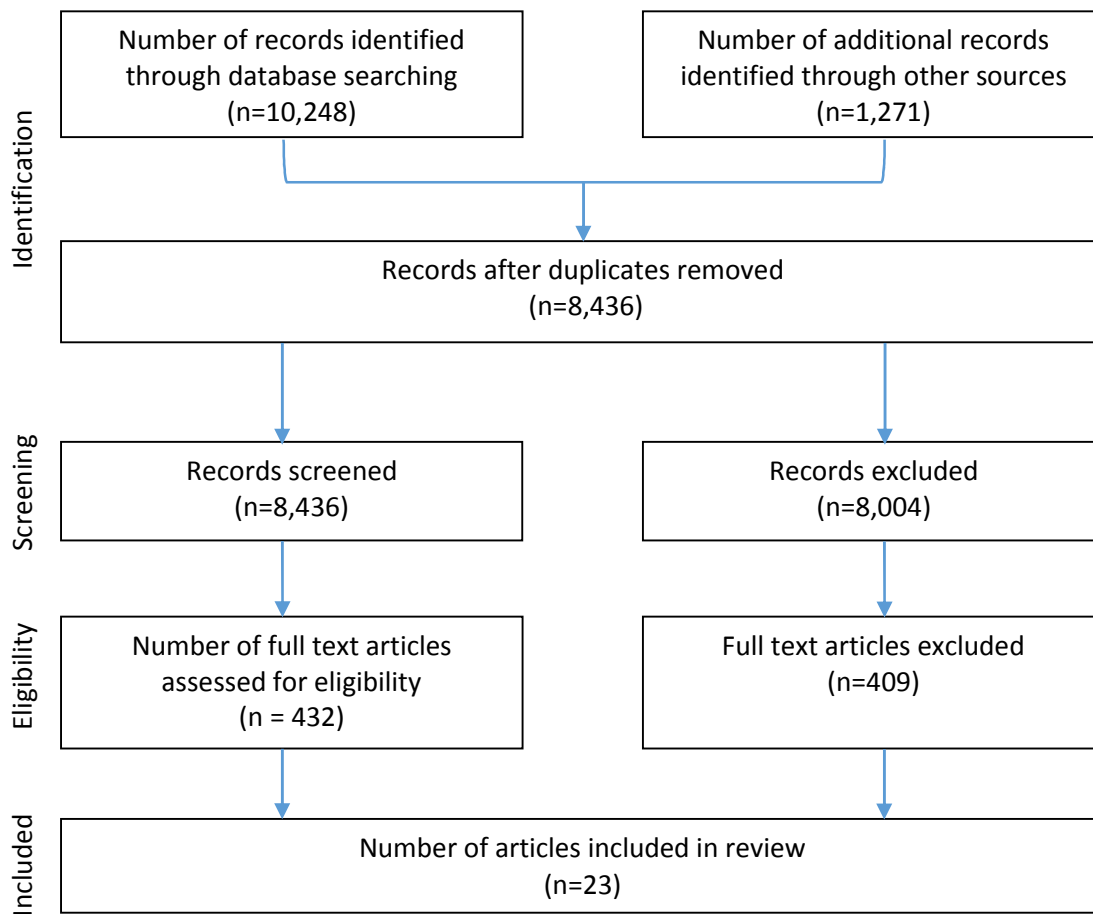

Figure 1. Review flowchart

Supplement: Supplementary data [file bmjsem-2017-000231supp003.pdf]
